# Lycium‐Barbarum Polysaccharide‐Loaded Dual‐Crosslinked Rigid Hydrogel Enhances Bone Healing in Diabetic Bone Defects by Scavenging Reactive Oxygen Species

**DOI:** 10.1002/adhm.202404741

**Published:** 2025-03-17

**Authors:** Wenjie Zhong, Wenao Liao, Lingcong Xu, Niezhenghao He, Ke Xu, Caiyuan Liu, Fei Wang, Wei Zhang, Jiang Hu, Haowen Cui

**Affiliations:** ^1^ Department of Orthopedics Sichuan Provincial People's Hospital School of Medicine University of Electronic Science and Technology of China Chengdu 610072 China

**Keywords:** bone defects, bone regeneration, diabetes, hydrogels, lycium barbarum polysaccharide

## Abstract

Diabetes‐induced oxidative stress can lead to poor bone defect healing, severely affecting the quality of life for patients. Studies show that improving the microenvironment and promoting bone formation can effectively accelerate the healing of bone defects. However, traditional local drug delivery methods face various challenges during the treatment process. Therefore, this study develops a novel hydrogel (HLBP) loaded with natural protein polysaccharides (LBP) extracted from goji berries, aiming to enhance the healing of diabetic bone defects. The hydrogel is composed of freeze‐dried polyvinyl alcohol (PVA) and photocrosslinked poly (ethylene glycol) diacrylate (PEGDA). This hydrogel exhibits excellent biocompatibility. Additionally, it demonstrates effective loading capacity for the LBP. LBP's bioactivity enables ROS scavenging and promotes bone regeneration at defect sites. In vitro, experimental results show that HLBP significantly reduces ROS levels and enhances osteogenic differentiation ability and cell viability of human bone marrow mesenchymal stem cells. In vivo studies using BKS‐db diabetic mice show that HLBP implantation at bone defects achieves over 80% healing, highlighting its strong healing potential. This method effectively avoids potential toxicity from systemic drug administration and significantly promotes regeneration at the bone defect site, providing a new strategy for treating diabetic bone defects.

## Graphical abstract

A novel hydrogel (HLBP) incorporating natural polysaccharides from goji berries is developed to enhance diabetic bone defect healing. The hydrogel efficiently scavenges ROS, improves osteogenic differentiation, and promotes in vivo bone regeneration. This strategy offers a biocompatible and localized approach, overcoming the challenges of traditional drug delivery in diabetic bone repair.

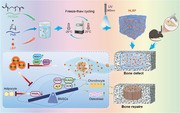

## 1 Introduction

Diabetes presents a global health concern, and individuals with diabetes often experience delayed or inadequate bone healing following fractures, infection, tumors, or surgery. This leads to heightened pain, additional surgical procedures, psychological distress, and increased medical expenses.^[^ ^1^ ^]^ This may be related to the excessive glucose environment and sustained high levels of reactive oxygen species (ROS) after tissue damage,^[^ ^2^ ^]^ which further disrupt the osteogenic microenvironment, as high blood sugar inside cells can also enhance the generation of ROS in mitochondria,^[^ ^3^ ^]^ and these ROS directly interfere with the bone healing process. The elevation of ROS levels not only directly inhibits the activity of BMSC cells, interferes with the normal expression of osteogenic markers such as alkaline phosphatase (ALP), Osteocalcin (OCN), Runt‐related transcription factor 2 (RUNX_2_), but also induces apoptosis in bone progenitor cells and osteoblasts related to bone regeneration. At the same time, it promotes the differentiation of BMSCs from an osteogenic phenotype to an adipogenic phenotype.^[^ ^4^, ^5^, ^6^ ^]^ High levels of ROS can enhance osteoclast proliferation and activity through the regulation of Protein Kinase B (PKB)/AKT Signaling Pathway (AKT) and extracellular regulated protein kinases (ERK), thereby extending the bone healing process.^[^ ^7^ ^]^ The generation of bone tissue in the body, which is a finely regulated process dominated by osteogenic precursor cells and osteoblasts, is significantly disrupted by ROS, affecting the conditions necessary for osteogenesis.^[^ ^8^ ^]^ Restoring a favorable osteogenic microenvironment by reducing elevated levels of ROS and mitigating chronic inflammation during the bone defect healing process is crucial for promoting bone regeneration in the context of diabetes.^[^ ^9^ ^]^

To facilitate the healing of bone defects, various materials have been developed, such as different biological membranes, autogenous bone, and bone paste.^[^ ^10^, ^11^, ^12^, ^13^ ^]^ However, the constrained plasticity and specific shape of biological membranes present challenges for their direct application in orthopedic surgery. Autologous bone transplantation requires the creation of two surgical sites (donor and recipient areas), which increases the complexity and trauma of the surgery, as well as the surgical time. Meanwhile, despite the favorable mechanical properties and low immune response of allogeneic bone, the unique cultural practice of burial in China has resulted in a significant shortage, as evidenced by the National Health Commission's data indicating an organ donation rate of 3.98 per million people in 2022. Many doctors face the challenge of having no bone available during clinical surgeries. Currently, various materials are used in clinical practice for bone defect repair, such as absorbable artificial bone powder primarily made from bioactive bio‐oxidized hydroxyapatite, collagen scaffolds made from type I or II collagen, and strip‐like artificial bone made from calcium sulfate. However, these materials all have limitations, including high costs and the need for long‐term clinical validation. Most materials are unable to completely satisfy the requirements for bone repair due to their limitations.

Hydrogels have been widely recognized as the future direction of tissue engineering due to their structural similarity to the extracellular matrix and excellent biocompatibility. For example, Chen et al. integrated MMP‐sensitive peptides containing chiral amino acids with norbornene (NB)‐modified 8‐arm polyethylene glycol (PEG) macromers to form a hydrogel network, which was demonstrated to effectively eliminate ROS and promote bone regeneration in a mouse skull defect model.^[^ ^14^ ^]^ Similarly, Mao et al. engineered mechanically robust silk fibroin/magnesium (SF/MgO) composite scaffolds to facilitate bone defect regeneration in the same model.^[^ ^15^ ^]^ To enhance bone repair efficacy, materials capable of delivering drugs or factors that promote regeneration have been engineered. Hydrogels, with their inherent hydrophilicity, facilitate versatile drug delivery systems featuring flexible administration routes and high drug loading capacities, thereby improving the microenvironment for osteogenesis. Wang et al. enhanced the drug loading capacity and prolonged the drug release duration by incorporating 43 wt.% vancomycin into a gelatin matrix, ultimately achieving the function of infection prevention during the bone repair process.^[^ ^16^ ^]^ However, hydrogel delivery systems have not been sufficiently validated under diabetic conditions, leading to significant shortcomings in the application of bone repair materials in high‐glucose environments. Therefore, developing a hydrogel capable of effectively eliminating ROS, improving the microenvironment, and promoting osteogenesis may be a promising solution to this issue.

The Lycium barbarum polysaccharide (LBP) from Ningxia was a natural antioxidant derived from plants, renowned for its elevated safety profile and properties including anti‐oxidative, anti‐inflammatory effects, as well as the promotion of osteoblast proliferation and differentiation.^[^ ^17^, ^18^, ^19^ ^]^ LBP corrected autophagic damage in human umbilical vein endothelial cells (HUVEC),^[^ ^20^ ^]^ regulated oxidative stress and ferroptosis via the nuclear factor erythroid 2‐related factor 2 (NRF2) pathway,^[^ ^21^ ^]^ and promoted osteogenesis of human periodontal ligament stem cells (hPDLSCs) through the ERK pathway.^[^ ^22^ ^]^ LBP not only inhibits hypoxia/reoxygenation (H/R)‐induced cardiomyocyte (H9C2) apoptosis through the nuclear factor E2‐related factor 2/heme oxygenase‐1 (Nrf2/HO‐1) signaling pathway,^[^ ^23^ ^]^ but also alleviates impaired glucose tolerance in type 2 diabetic KKAy mice (IGT‐KKAy) by suppressing the expression of sodium/glucose cotransporter 1 (SGLT1) and increasing the release of glucagon‐like peptide 1 (GPL1).^[^ ^24^ ^]^ LBP, which effectively eliminates ROS and promotes osteogenesis, has garnered our attention. However, LBP is rapidly metabolized in vivo, requiring long‐term administration by patients. Therefore, it is essential to develop platforms that can enhance drug delivery efficiency and extend the duration of its action to maximize its therapeutic potential.

In this investigation, we developed a porous hydrogel with dual physical and chemical crosslinking networks loaded with LBP (HLBP), which exhibits high mechanical stability, excellent biocompatibility, and remarkable shape adaptability. In vivo experiments confirmed that HLBP10 (the HLBP hydrogel with a 10% mass fraction) promotes bone regeneration at the defect site and efficiently repairs the defect in situ. This study underscores the effectiveness of hydrogels loaded with drug molecules for repairing diabetic bone defects.

## 2 Results

### 2.1 Synthesis and Characterization of the HLBP Hydrogel

**Figure 1:**
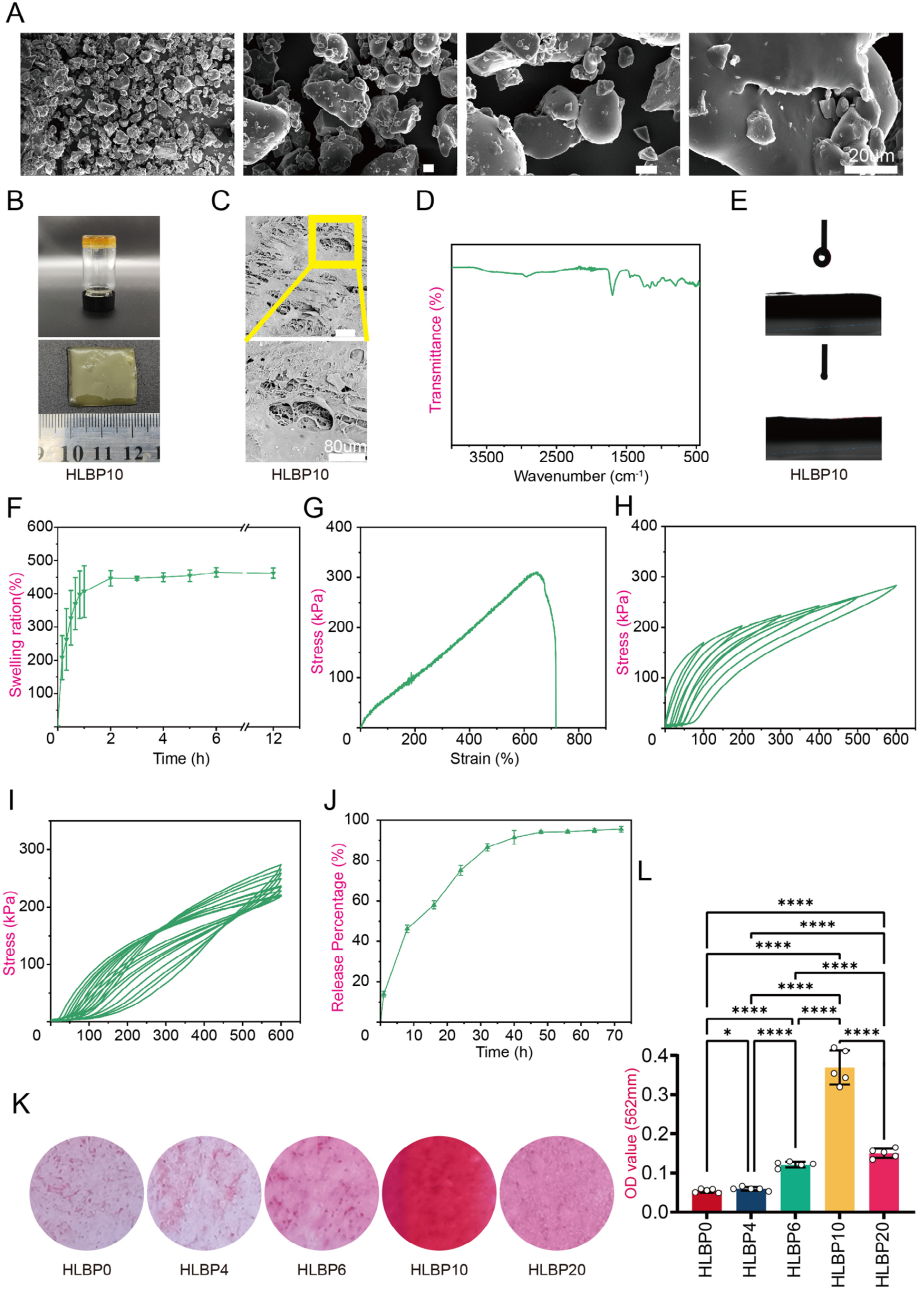
Characterization of HLBP hydrogel. A) Images of LBP under SEM at different magnifications (scale bar = 20 µm). B) Physical image of HLBP10. C) SEM image of HLBP10 (scale bar = 80 µm). D) FTIR analysis of HLBP10 hydrogel (*n* = 3). E) Water contact angle image of HLBP10. F) Swelling test analysis of HLBP10 hydrogel (*n* = 3). G) Tensile test analysis of HLBP10 hydrogel (*n *= 3). H) Gradient Strain Loading–Unloading Test of HLBP10 Hydrogel (*n* = 3). I) Cyclic Loading–Unloading Test of HLBP10 Hydrogel (*n* = 3). J) The LBP release curve of HLBP10 hydrogel (*n* = 3). K) ARS staining of hBMSC cells co‐cultured with different masses of loaded HLBP. L) Quantitative analysis results of Alizarin Red staining (*n* = 5). Data are presented as mean ± SD, *p < 0.05, **p < 0.01, ***p < 0.001. (HLBP0 group is the co‐culture group of HLBP extract with a mass fraction of 0; HLBP6 is the co‐culture group of HLBP extract with a mass fraction of 6; HLBP10 group is the co‐culture group of HLBP extract with a mass fraction of 10; HLBP20 is the co‐culture group of HLBP extract with a mass fraction of 20).

In order to prepare hydrogel materials with strong mechanical stability, superior biocompatibility, and excellent shape adaptability, we selected PVA as one of the raw materials due to its low cytotoxicity and FDA approval for oral administration. PEGDA and Acrylic acid were also chosen for its excellent hydrophilicity, biocompatibility, and photo‐controllability. To circumvent the use of potentially toxic crosslinking agents and ensure the mechanical integrity of the material, we employed a double network (DN) approach for hydrogel preparation. The physical crosslinking phase of PVA was formed through continuous freeze‐drying while the chemical crosslinking structure was formed under UV light exposure, ensuring the mechanical properties of the material. The morphology of dried LBP and HLBP were observed using scanning electron microscopy (SEM). As shown in Figure (**Figure** **1**A), LBP appeared granular with a relatively smooth surface and slight wrinkles without obvious agglomeration. In contrast, after the introduction and preparation of HLBP (Figure 1B; Figure S1, Supporting Information), the composite hydrogel without LBP exhibited a smooth surface and a uniform porous structure at the micron scale. When a small amount of LBP was added, the surface showed particle precipitation. However, with the introduction of a higher mass fraction of goji berry polysaccharides, the surface developed oily substances similar to soluble goji polysaccharides, resulting in slightly larger pore sizes and an uneven distribution (Figure 1C; Figure S2, Supporting Information).

Additionally, the chemical composition and secondary structure content of the HLBP were analyzed using FTIR, as depicted in Figure (Figure 1D; Figure S3, Supporting Information). The infrared spectrum of LBP exhibits a characteristic broad peak corresponding to the hydroxyl group at 3307 cm^−1^, and the C–H stretching vibration peak is observed at 2925 cm^−1^. The peak at 1630 cm^−1^ may be attributed to the stretching vibration of free water or C═O.^[^ ^25^ ^]^ The band observed at 1000 cm^−1^ is indicative of the β‐configuration present in polysaccharides.^[^ ^26^ ^]^ The HLBP0 vibration peak occurs at ≈3300 cm^−1^, and the polymer C–O stretching vibration peak is observed ≈1150 cm^−1^. Furthermore, due to the overlapping peak positions, only HLBP20 was able to observe the β‐configuration (1000 cm^−1^) and hydroxyl peak (3307 cm^−1^) of LBP, with no additional characteristic peaks of LBP being detected.

### 2.2 The Mechanical and Multifunctional Properties of HLBP

Excellent hydrophilicity is a crucial factor in facilitating fluid exchange, promoting cell proliferation, and enhancing the early stages of bone regeneration.^[^ ^27^ ^]^ We conducted water contact angle evaluations of HLBP with various mass fractions (Figure 1E; Figure S4, Supporting Information) and observed favorable water contact angles for all HLBP samples. Additionally, we performed swelling experiments to assess the hydrophilicity of HLBP. In the PBS swelling experiment at 37 °C, all mass fractions of HLBP reached swelling equilibrium within only 2 h (Figure 1F; Figure S5, Supporting Information), as depicted in the figures, an increase in filler content led to a rise in the swelling ratio of HLBP from 283 ± 13% to 516.8 ± 22.5%, demonstrating that LBP effectively enhances the hydrophilicity of HLBP.

The mechanical stability of HLBP has been a focus of our attention. It has been reported in the literature that hydrogels prepared based on double interpenetrating networks exhibit favorable mechanical properties. The HLBP developed in this study utilizes the physical cross‐linking network of PVA and the photo‐cross‐linking network of PEGDA and Acrylic acid to form a dense structure, ensuring its resistance to external forces post‐implantation. Tensile performance tests conducted on different HLBP samples indicate that the incorporation of an appropriate amount of LBP did not significantly alter their tensile properties. The tensile strength of HLBP ranges from 0.24–0.36 MPa, with an elongation at break ranging from 691.8–781% (Figure 1G; Figure S6, Table S1, Supporting Information). For bone defect scaffolds, hydrogels must withstand prolonged loading, which may lead to fatigue over time. To address this issue, we designed a double‐network hydrogel to ensure mechanical stability. Similar structures have been widely reported in the literature. For instance, Gu et al. developed an interpenetrating PVA/PEGDA network with a comparable design, demonstrating excellent mechanical properties.^[^ ^28^ ^]^ We evaluated the fatigue resistance of our hydrogel through gradient strain loading–unloading tests and cyclic loading–unloading tests. The experimental results indicate that the PVA‐dominated gel network acts as a sacrificial phase, breaking preferentially, while the photocrosslinked chemical network further dissipates stress and prevents crack propagation. The destruction of the physically crosslinked network, along with the deformation and fracture of the chemically crosslinked network, contributes to effective energy dissipation (Figure 1H; Figure S7, Supporting Information). A significant amount of energy was dissipated in the first cycle, while subsequent cycles exhibited lower energy dissipation and tensile strength. This suggests that the covalently crosslinked network was damaged and could not recover or reconstruct (Figure 1I; Figure S8, Supporting Information), supporting the conclusion that our material exhibits superior fatigue resistance.

Despite this, the hydrogel maintained its structural integrity throughout ten cycles of the loading–unloading test, demonstrating good fatigue resistance.

The degradation experiment shows the remaining mass percentage of HLBP with different mass fractions after immersion in a 37 °C culture medium (Figure S9, Supporting Information). It is evident that, following a 3‐day immersion period, the mass loss closely aligns with the introduced LBP content. This indicates that, as per the extraction method outlined in GB/T 16886.5‐2017, the concentration of LBP in the extract of different materials is ≈0.8, 1.5, 2, and 4 mg mL^−1^. Additionally, the physicochemical properties of HLBP at various mass fractions were stable. HLBP hydrogel also exhibited good drug loading and release capabilities, gradually releasing LBP within 8‐hour intervals (Figure 1J; Figure S12, Supporting Information). Specifically, the release slowed down and stabilized after 60 h. The final drug loading rates for each group were 0.37%, 0.56%, 0.94%, and 1.8%, respectively.

Therefore, different mass fractions of HLBP extracts were used in in vitro experiments to determine the optimal concentration for promoting osteogenic differentiation of BMSCs (Figure 1K,L). The results indicated that under extreme sugar culture conditions, HLBP10 was the most effective in enhancing the osteogenic differentiation of BMSCs.

### 2.3 Biocompatibility of the HLBP Hydrogel

**Figure 2:**
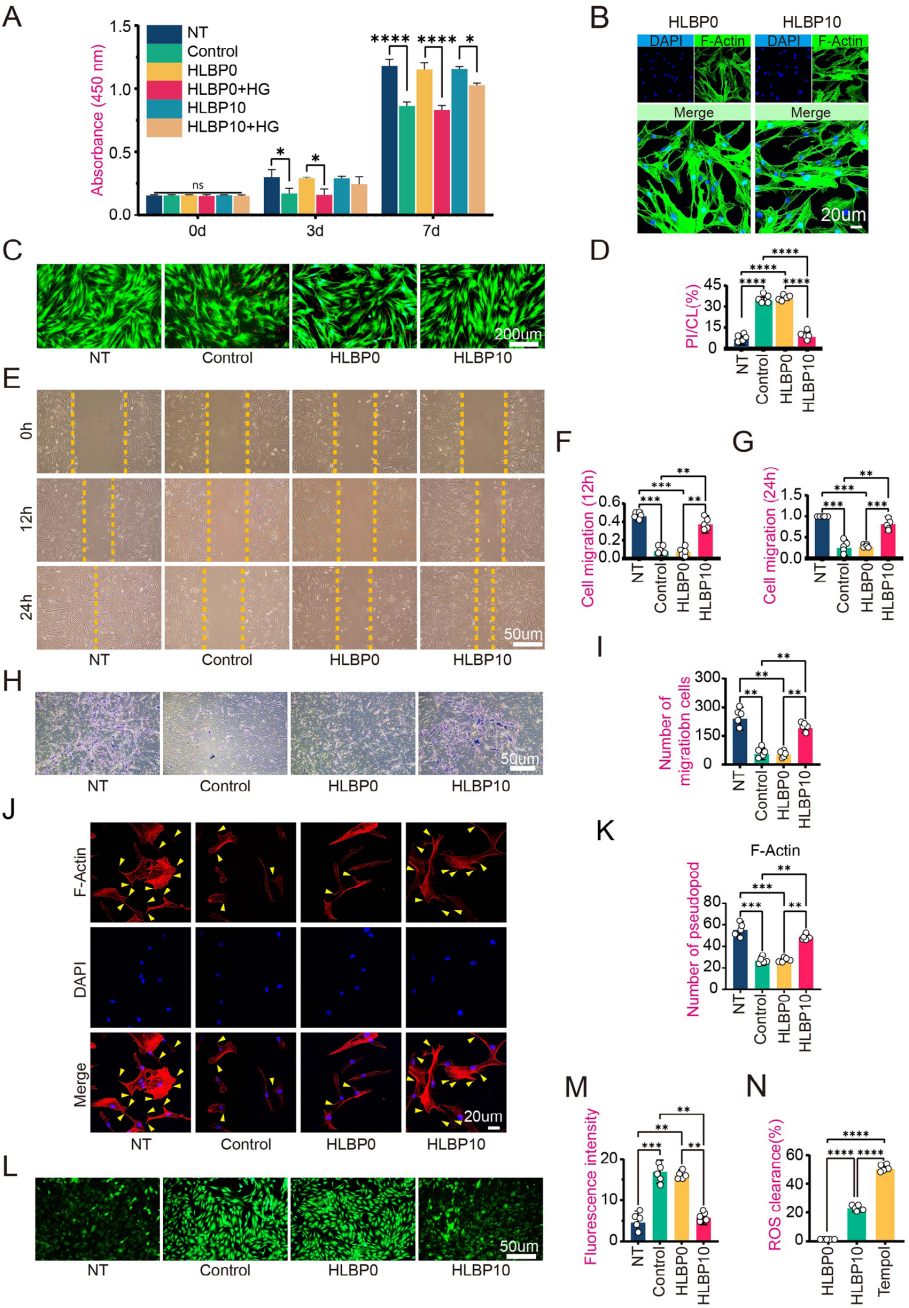
The effect of HLBP10 hydrogel on the viability and migration ability of BMSC cells under in vitro oxidative stress. A) CCK8 activity assay of BMSCs under different culture conditions. B) Cell morphology staining (F‐Actin) of BMSCs on HLBP. C) Staining of live/dead cells in each group. D) Quantitative analysis of the staining results for each group's viability/death. (Calcein AM stains live cells, showing green fluorescence; while Propidium Iodide (PI) stains dead cells, showing red fluorescence.) E) The scratch experiment results of BMSCs in each group. F) Quantitative analysis of the migration ability of BMSCs in each group at 12 h. G) Quantitative analysis of the migration ability of BMSCs in each group at 24 h. H) The Transwell experiment results of each group of BMSCs. I) Quantitative analysis of the Transwell experiment results of BMSCs in each group. J) The cell morphology staining (F‐Actin) of BMSCs from different groups was compared based on the number of pseudopods. K) Analysis of the number of pseudopods in BMSCs from different groups. L) Measurement of ROS levels in BMSCs from different groups after treatment by various methods. M) Quantitative analysis of the ROS levels in BMSCs from different groups after being treated in different ways. N) Determination of the free radical scavenging ability of HLBP10 was carried out using the DPPH method. Data are presented as mean ± SD, *n *= 5, *p < 0.05, **p < 0.01, ***p < 0.001. (The NT group was cultured in a regular medium, the Control group was cultured in a high‐sugar medium, the HLBP0 group was cultured with HLBP extract at a mass fraction of 0, and the HLBP10 group was cultured with HLBP extract at a mass fraction of 10).

The effects of HLBP hydrogel on the proliferation and viability of human bone marrow mesenchymal stem cell (hBMSC) cells were verified through CCK‐8 analysis. The CCK8 results indicated no statistically significant differences in cell viability and optical density (OD) values across the experimental groups (**Figure** **2**A). The cells in both the HLBP10 group and the HLBP0 group, as shown by F‐actin immunofluorescence staining, exhibited enhanced migratory behavior (Figure 2B). The results demonstrated that the hydrogel materials exhibited excellent biocompatibility.

### 2.4 HLBP Hydrogel can Remove ROS and Improve Cell Vitality and Migration

Under hyperglycemic conditions in diabetes, a high level of ROS is generated, creating a microenvironment that reduces cell viability and osteogenic function, leading to impaired bone defect healing.^[^ ^29^ ^]^ We conducted preliminary research using a high‐sugar culture medium to simulate an in vitro hyperglycemic environment.^[^ ^30^ ^]^ We designated the group using the regular medium as the NT group. The group using only high‐glucose medium was set as the Control group. The group with added HLBP0 hydrogel in the high‐glucose medium was designated as the HLBP0 group, and the group with added HLBP10 hydrogel in the high‐glucose medium was designated as the HLBP10 group. Under elevated‐sugar culture conditions, both live/dead fluorescence images and CCK8 assays revealed significantly superior viability of the HLBP10 group compared to the HLBP0 and Control groups (Figure 2A,C,D). The optical density (OD) value of the HLBP10 group is significantly higher than that of the HLBP0 and Control groups (Figure 2A), indicating that HLBP10 can maintain the cell viability of hBMSCs under high glucose conditions.

During the process of bone defect repair, the migration and aggregation of hBMSC cells are essential.^[^ ^31^ ^]^ The effect of HLBP10 on improving hBMSCs cell migration was evaluated through cell scratch experiment, Transwell experiment, and F‐Actin immunofluorescence staining (Figure 2E–K). In the scratch assay and Transwell experiment, the cell migration distance and the number of migrating cells in the HLBP10 group were closer to those of the NT group. Additionally, the IF results for F‐Actin showed that the HLBP10 group had a greater number of filopodia. This indicates enhanced motility of the BMSCs in this group. ROS was detected using a reactive oxygen species probe, and the results showed that HLBP10 treatment reduced ROS levels (Figure 2L,M). In addition, we used the DHHP method to detect HLBP0 and HLBP10 and found that the clearance rate in the HLBP10 group was higher (23 ± 1.4) (Figure 2N).

NRF2 is a transcription factor that plays a crucial role in cellular defense against oxidative stress. Under oxidative stress conditions, NRF2 protein expression increases significantly, leading to its translocation into the nucleus, where it binds to the antioxidant response element (ARE). This interaction promotes the transcription of ARE‐dependent genes, such as heme oxygenase‐1 (HO‐1) and NAD(P)H quinone oxidoreductase 1 (NQO1).^[^ ^32^ ^]^

It has been reported that LBP can enhance the expression and activity of NRF2, HO‐1, and NQO1 in oxidative stress environments.^[^ ^33^ ^]^ As shown in Figure (Figure S10A–J, Supporting Information), in a high‐glucose environment, the expression of NRF2 was reduced in the Control and HLBP0 groups, whereas the HLBP10 group exhibited a significant increase in NRF2 protein levels, even surpassing those in the NT group. Additionally, the expression levels of HO‐1 and NQO1 were lower in the Control and HLBP0 groups, while the HLBP10 group demonstrated marked upregulation of NRF2, HO‐1, and NQO1.

These results indicate that HLBP10 effectively reduces ROS levels and preserves the migration capability of hBMSCs.

### 2.5 The HLBP Hydrogel Enhances the Proliferation of hBMSC Cells in Hyperglycemic Conditions and Suppresses the Adipogenic Differentiation of hBMSC

**Figure 3:**
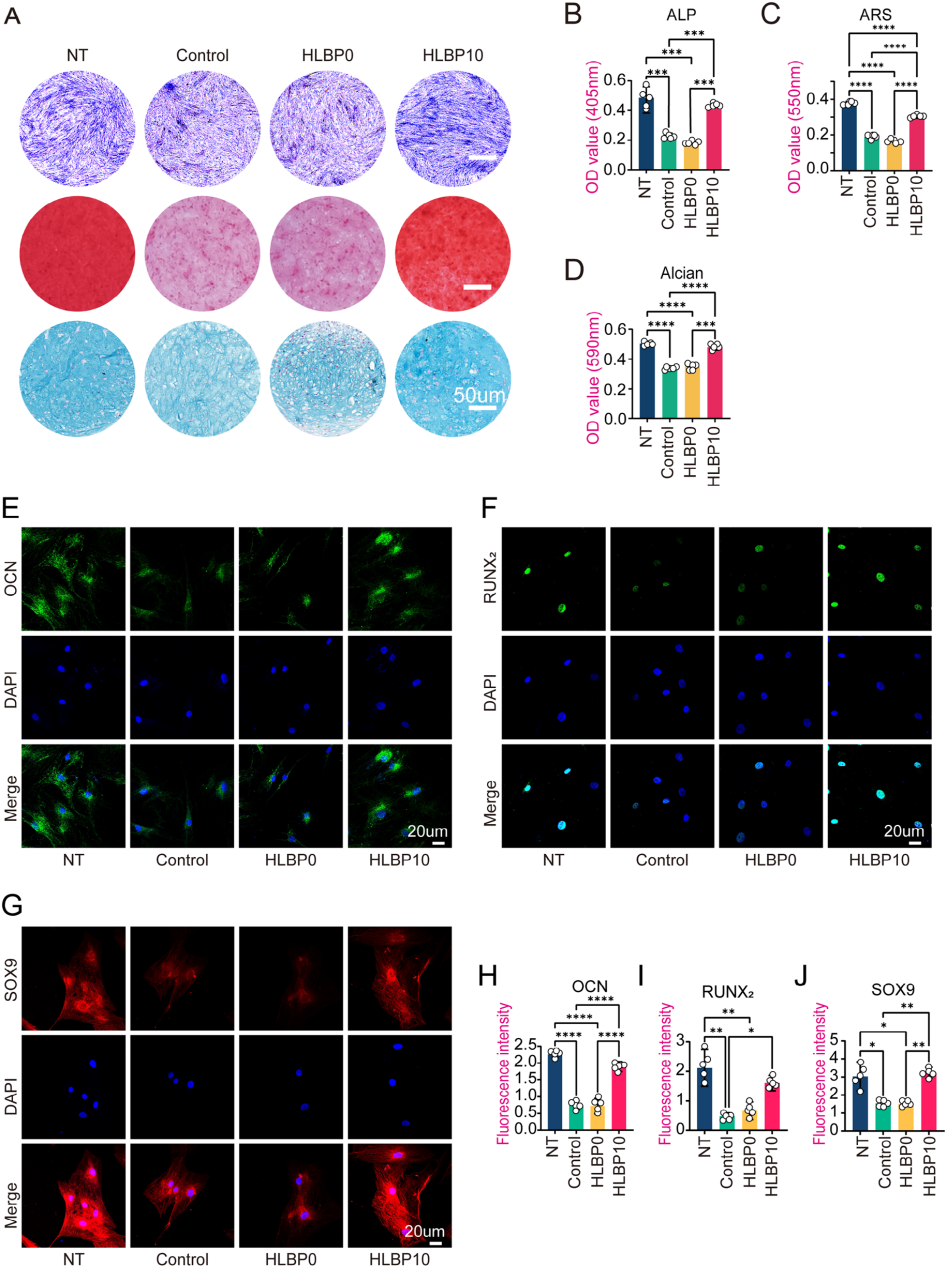
The influence of HLBP10 hydrogel on the differentiation ability of BMSC cells under in vitro oxidative stress. A) Different groups were subjected to alkaline phosphatase staining (ALP), alizarin red staining (ARS), and Alcian blue staining (AB) under different culture conditions. B) Quantitative analysis graph of alkaline phosphatase staining results. C) Quantitative analysis of the staining results of Alizarin Red. D) Quantitative analysis graph of the staining results of Alcian. E) Immunofluorescence staining of OCN antibody protein in different groups. F) Immunofluorescence staining of RUNX_2_ antibody protein in different groups. G) Immunofluorescence staining of SOX9 antibody protein in different groups. H–J) Quantitative analysis of OCN (RUNX_2_, SOX9) antibody protein cell immune fluorescence staining results. Data are presented as mean ± SD, *n* = 5, *p < 0.05, **p < 0.01, ***p < 0.001. (The NT group was cultured in a regular medium, the Control group was cultured in a high‐sugar medium, the HLBP0 group was cultured with HLBP extract at a mass fraction of 0, and the HLBP10 group was cultured with HLBP extract at a mass fraction of 10).

**Figure 4:**
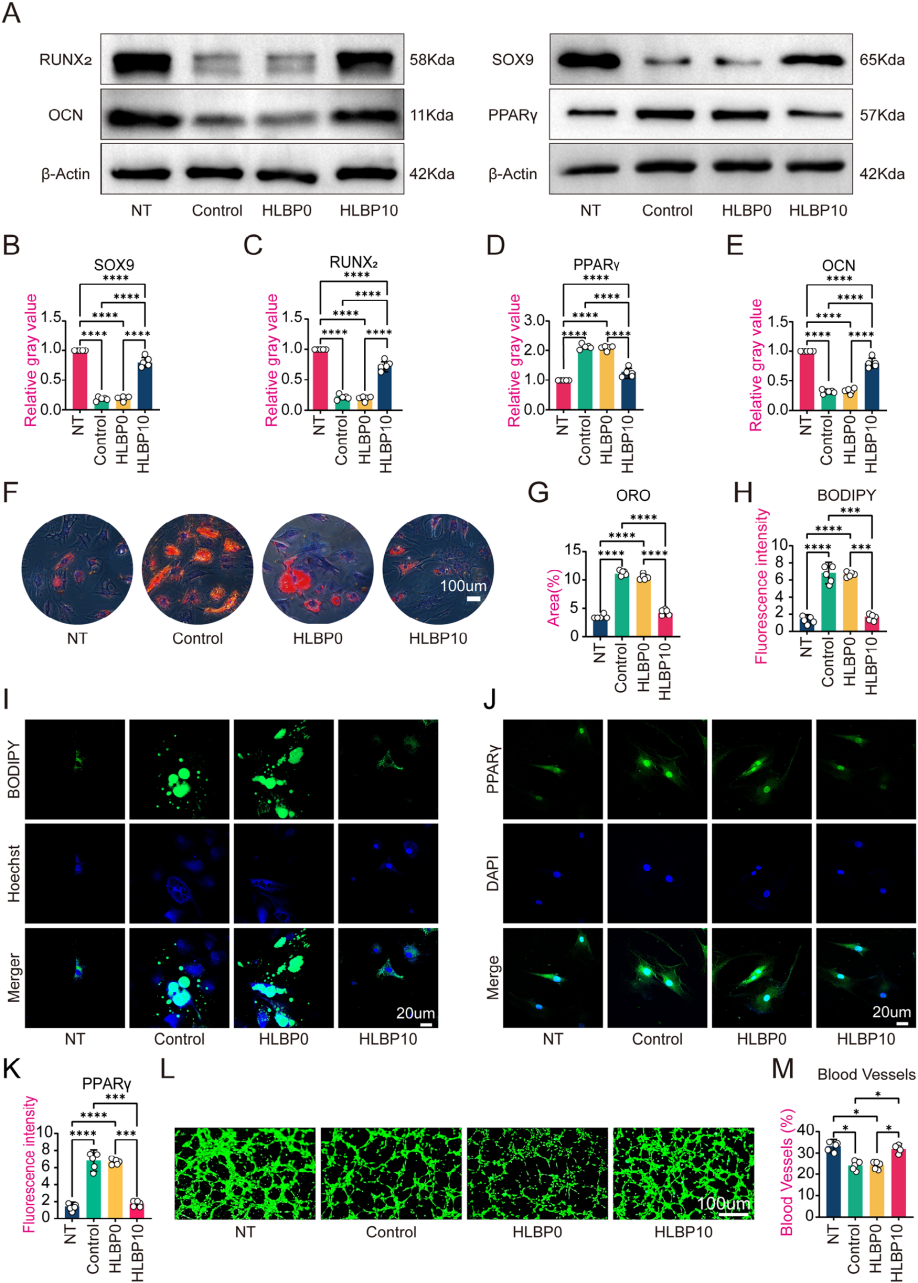
The influence of HLBP10 hydrogel on the differentiation ability of BMSC cells under in vitro oxidative stress. A) Protein imprint (WB) analysis of SOX9, RUNX_2_, PPARγ, and OCN proteins in each group. B–E) Quantitative results of WB analysis for SOX9, RUNX_2_, PPARγ, and OCN proteins in each group. F) The oil red O (ORO) staining results of each group. G) Quantitative analysis of the Oil Red O (ORO) staining results for each group. H) Quantitative analysis of the BODIPY staining results for each group. I) The BODIPY staining results for each group. J) The cell immunofluorescence staining results of PPARγ protein in each group. K) Quantitative analysis of cell immunofluorescence in Figure 4J. L) Experimental results of HUVEC cells forming blood vessels under each culture condition. M) Quantitative analysis of the blood vessel formation experiment in HUVEC cells of Figure 4K. Data are presented as mean ± SD, *n* = 5, *p < 0.05, **p < 0.01, ***p < 0.001. (The NT group was cultured in a regular medium, the Control group was cultured in a high‐sugar medium, the HLBP0 group was cultured with HLBP extract at a mass fraction of 0, and the HLBP10 group was cultured with HLBP extract at a mass fraction of 10).

In a high blood sugar environment, the osteogenic ability of hBMSC cells decreases and they are more prone to differentiate into a fat phenotype, which is closely related to the difficulty in healing bone defects in diabetes‐related diseases.^[^ ^34^ ^]^ ALP and ARS staining were used to assess the early osteogenic performance and mineralization capacity of hBMSCs under high‐glucose conditions (**Figure** **3**A–C). The HLBP10 group exhibited significantly superior results compared to the Control group. Subsequently, AB staining was employed to assess the chondrogenic differentiation capacity of hBMSC, revealing deeper staining in the cell pellet sections of the HLBP group compared to both the HLBP0 group and Control group (Figure 3A,D). We also utilized ORO to assess the adipogenic function of hBMSC. It was observed that both the Control group and HLBP0 group exhibited a darker color and larger area of lipid droplets, leading to distorted and shrunken cell morphology due to the accumulation of lipid droplets (**Figure** **4**F,G). In order to observe and quantify lipid droplets more intuitively, we also used the BODIPY staining method to fluorescently stain the lipid droplets (Figure 4H,I). The results showed similar trends, as confirmed by subsequent IF (Figures 3E‐–J;4J,K) and Western blot (Figure 4A–E) analysis, indicating consistency with the previous findings in both qualitative and quantitative aspects. These findings collectively suggest that hBMSC exhibit a reduced tendency to differentiate into osteogenic and chondrogenic phenotypes under high‐sugar culture conditions, while their adipogenic differentiation propensity is enhanced. However, HLBP10 has the ability to counteract these tendencies and preserve the osteogenic and chondrogenic phenotypes of hBMSC, thereby promoting bone regeneration.

During the process of bone regeneration, the formation of small blood vessels plays a crucial role in providing essential nutrients and creating optimal oxygen conditions to accelerate local metabolism.^[^ ^8^ ^]^ Therefore, we conducted tests on the angiogenic ability of HUVEC cells in a high‐sugar environment (Figure 4L,M). The results demonstrated that the vascular area in the HLBP10 group was significantly greater than that in the Control group and HLBP0 group, indicating that HLBP10 can promote HUVEC cells to generate vascular networks more quickly and abundantly in a high‐sugar environment.

### 2.6 HLBP10 Helps Reduce the Production of ROS in Diabetic Bone Defect Sites

**Figure 5:**
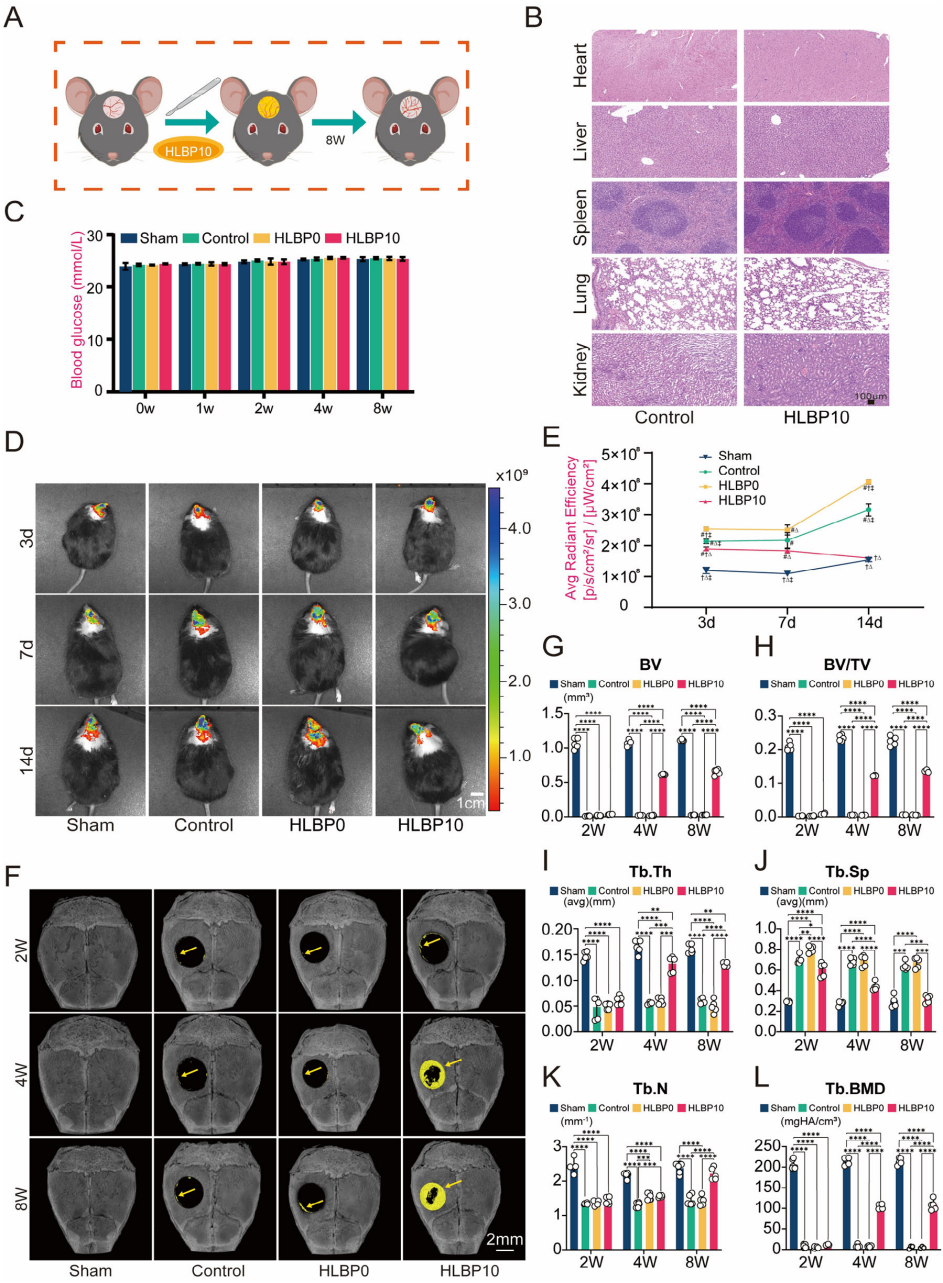
Clearance of ROS and bone regeneration assessment in diabetic bone defects. A) Schematic diagram of implanting ROS‐responsive biodegradable hydrogel to repair diabetic bone defects. B) Analysis of the impact on important organs after HLBP10 hydrogel implantation. C) Blood glucose monitoring in diabetic mice. D) Representative fluorescence images of ROS clearance at different time points in diabetic mice. E) Quantitative analysis of representative fluorescence images of ROS clearance at different time points in diabetic mice. (#: indicates a significant difference compared to the Sham group; †: indicates a significant difference compared to the Control group; ∆: indicates a significant difference compared to the HLBP0 group; ‡: indicates a significant difference compared to the HLBP10 group.) F) Representative 3D reconstructed micro‐CT images of skull defects at 2, 4, and 8 weeks after different treatments. G–L) Analysis graphs for bone regeneration parameters including BV (bone volume), BV/TV (bone volume/tissue volume), Tb.Th (trabecular thickness), Tb.Sp(trabecular separation), Tb.N(trabecular number), and Tb.BMD(bone mineral density) in the skull defect area at 2, 4, and 8 weeks after different treatments. Data are presented as mean ± SD, *p < 0.05, **p < 0.01, ***p < 0.001. (The Sham group is a mouse model group in which only skin incisions are made; the Control group is a mouse model group in which bone defects are created but not filled with HLBP treatment; the HLBP0 group is a mouse model group with bone defects filled with HLBP treatment at a mass fraction of 0; and the HLBP10 group is a mouse model group with bone defects filled with HLBP treatment at a mass fraction of 10. The number of mice in each group was *n* = 5).

We performed a 3.0 mm circular cranial defect surgery on 6‐8‐week‐old male diabetic mice (bk‐db) to establish a bone defect model (**Figure** **5**A). At week 8 post‐implantation of the corresponding materials, HE staining was conducted on major organs (heart, liver, spleen, lungs, kidneys) of mice from the Sham and HLBP10 groups to assess potential damage (Figure 5B). Additionally, the blood glucose levels of the mice were continuously monitored throughout the experiment (Figure 5C). The results showed that blood glucose levels remained consistently high during the experiment, and implantation of HLBP10 hydrogel did not cause any damage to the major organs. The specific ROS levels at the defect site after HLBP10 implantation need to be visualized and quantified using a fluorescent probe DCFH‐DA and an in vivo imaging system (IVIS). Live imaging revealed that on postoperative day 3, the ROS levels at the bone defects of all groups of mice were relatively high, slightly decreased on postoperative day 7, and then on postoperative day 14, there was a significant increase in ROS levels in Sham group, Control group, and HLBP0 group; conversely, on day 14, the ROS level in HLBP10 group further decreased (Figure 5D,E). This may be related to the timely reduction of ROS levels in the early stage of bone defects effectively preventing further damage to surrounding tissues caused by excessively high ROS levels, as well as inhibition of elevated ROS levels at damaged sites due to high blood glucose in diabetic mice themselves. At the same time, the polymer material gradually degrades in the body into low molecular weight compounds, which are then metabolized and excreted through organs such as the kidneys and liver.^[^ ^35^ ^]^ These results suggest that HLBP hydrogel demonstrates good biocompatibility and biosafety. Overall, we are of the opinion that HLBP10 decreased the capacity for ROS production at the site of bone defects in diabetic mice.

### 2.7 HLBP10 Helps to Increase Bone Regeneration at the Site of Diabetic Bone Defects

**Figure 6:**
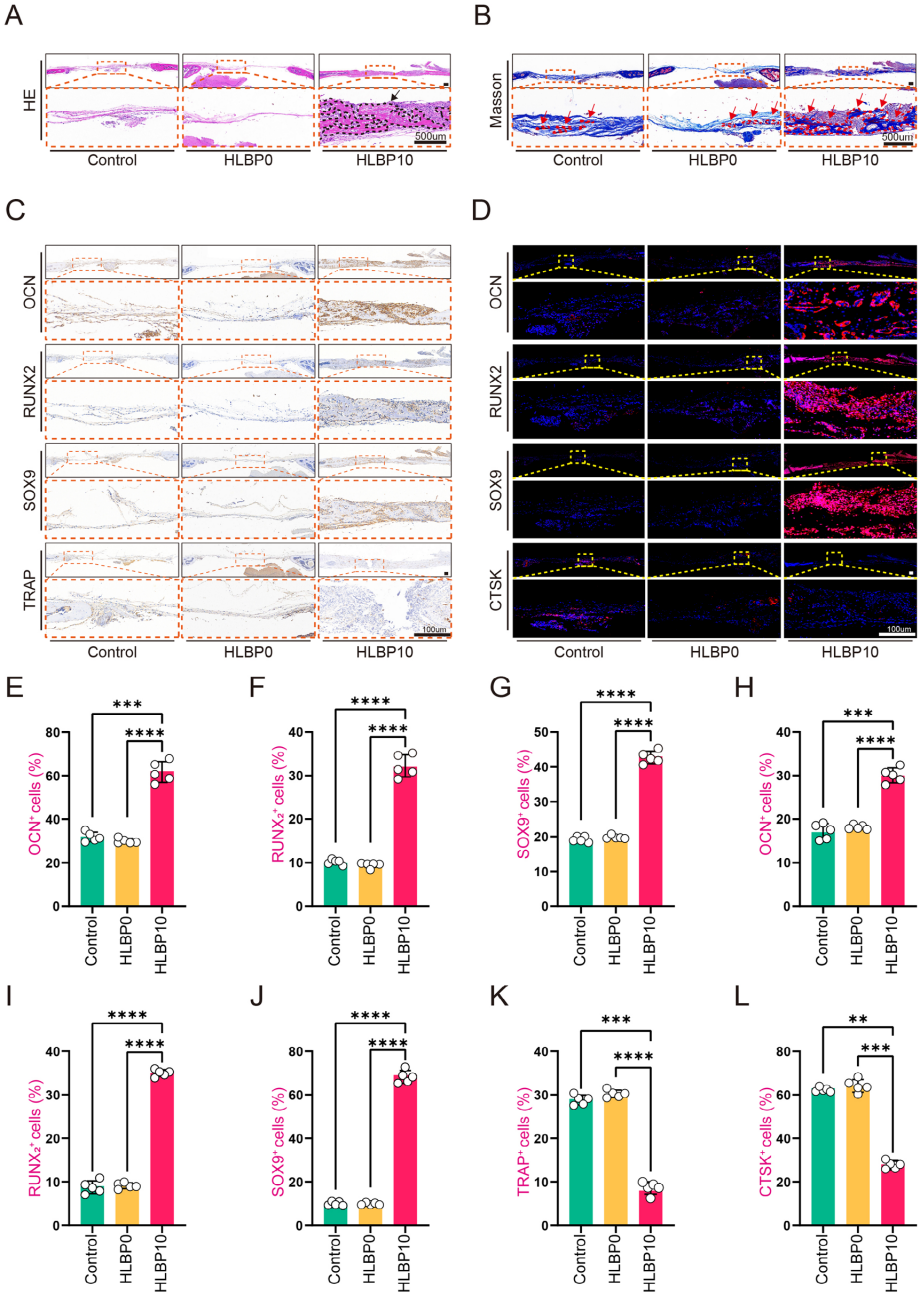
Different histological evaluations of diabetic bone defect tissues after treatment. A) H&E staining images of diabetic bone defect tissues. B) Masson staining images of diabetic bone defect tissues. C) Representative immunohistochemical staining images of osteocalcin (OCN), runt‐related transcription factor 2 (RUNX_2_), SRY‐Box Transcription Factor 9 (SOX9), and tartrate‐resistant acid phosphatase (TRAP). D) Representative immunofluorescence staining images of OCN, RUNX_2_, SOX9, and cathepsin K (CTSK). E–L) Quantitative analysis of immunohistochemical or immunofluorescence staining for OCN, RUNX_2_, SOX9, TRAP, and CTSK in the diabetic bone defect tissues. Data are presented as mean ± SD, Data are presented as mean ± SD, *n *= 5, *p < 0.05, **p < 0.01, ***p < 0.001.

To assess the osteogenic status at various time points, we utilized Micro‐CT for scanning and detecting the bone defect site at 2, 4, and 8 weeks postoperatively (Figure 5F–L). The 3D reconstruction images revealed minimal bone regeneration in the NT group and HLBP0 group mice, with only a small amount of new bone tissue present at 8 weeks postoperatively. In contrast, the HLBP10 group exhibited a significant amount of new bone tissue at both 4 and 8 weeks. Subsequently, HE staining (**Figure** **6**A) and Masson staining (Figure 6B) were performed on the skull bones of the HLBP10, HLBP0, and Control groups at 4 and 8 weeks. The HE staining results showed a higher incidence of new bone formation in the HLBP10 group at each time point, along with a significant reduction in the bone defect area. In contrast, both the HLBP0 and Control groups showed minimal healing. The results of immunohistochemistry and immunofluorescence (IF) in the bone defect region were consistent (Figure 6C–L), indicating that the HLBP10 group not only enhanced osteogenic activity at the bone defect site but also exhibited a certain inhibitory effect on osteoclast function. These findings suggest that HLBP has the potential to promote bone regeneration at diabetic bone defect sites. In conclusion, our study demonstrates that HLBP10 can enhance bone regeneration by modulating ROS levels at the bone defect site and promoting osteogenic protein expression.

## 3 Discussion

Diabetes mellitus (DM) is a complex endocrine and metabolic disorder that seriously affects human health, and the number of patients is gradually increasing. It is estimated that there will be over 629 million DM patients by 2045, with type 2 diabetes (T2DM) accounting for more than 90%.^[^ ^36^ ^]^ DM frequently leads to a range of severe complications, including diabetic nephropathy, cardiovascular and cerebrovascular diseases, neurological disorders, and diabetic bone disease. Additionally, alterations in the microenvironment due to factors such as high levels of ROS, elevated blood sugar, and microvascular lesions often result in delayed or non‐healing bone fractures in diabetic patients.^[^ ^37^ ^]^ Research has shown that treatment targeting ROS can effectively improve the microenvironment and help with bone healing at diabetic bone defect sites.^[^ ^29^, ^38^ ^]^

LBP, a natural protein‐polysaccharide extracted from goji berries, is characterized by its affordability and abundant availability. Ni et al. reduced the level of cellular ROS and protected retinal ganglion cells from iron‐induced death by activating the NRF2 pathway using LBP molecules, thus reducing neuroinflammation. Sun et al. investigated the effects of LBP on bone loss in adult mice, aging mice, and ovariectomized mice models, and found that LBP1C‐2 (a component of LBP) significantly increased bone mass and strength by directly binding to BMP receptors, promoting osteoblast proliferation, differentiation, and mineralization.^[^ ^33^, ^39^ ^]^ Zhu et al. also investigated the mechanism of LBP's anti‐inflammatory, anti‐apoptotic, and anti‐aging effects, demonstrating that LBP can protect skin cells from PM2.5‐induced cytotoxicity by regulating the oxidative stress‐ER stress‐autophagy‐cell apoptosis signaling axis, revealing its role in skin protection.^[^ ^40^ ^]^

Previous studies on LBP have typically used systemic administration for treatment, though some have employed localized delivery methods. Wang et al. innovatively loaded LBP onto nanofiber scaffolds constructed through electrospinning, leveraging LBP's ability to accelerate long‐distance axonal regeneration in peripheral nerves for the treatment of peripheral nerve injuries.^[^ ^41^ ^]^

To address the challenges of effectively delivering therapeutic agents for bone regeneration, we designed a dual‐network structured hydrogel as the carrier for HLBP. Specifically, the PVA hydrogel was prepared using a cyclic freeze–thaw method, which facilitated the formation of a 3D network through hydrogen bonding and microcrystalline regions. This physical cross‐linking approach avoids the use of chemical cross‐linkers, thereby ensuring the hydrogel's biocompatibility. The second network was established through the chemical cross‐linking of PEGDA and acrylic acid. Under photo‐initiation, the double bonds in the monomers formed a dense cross‐linked structure. The photoinitiator chosen for this process was α‐ketoglutaric acid, which has been reported to participate in the tricarboxylic acid cycle in vivo, contributing to biosynthesis and energy metabolism.^[^ ^42^ ^]^ The minimal concentration used in this system (0.2 w/w%) ensures the material's biocompatibility while maintaining structural integrity. First, we validated in the diabetes cell model that HLBP reduced cellular ROS levels and inhibited the tendency of hBMSC to differentiate into adipocytes, promoting osteogenic expression and ultimately facilitating the process of bone regeneration. This finding is similar to that of Zhang et al., who synthesized a factor‐free hydrogel made from thioketone (TK) and NB to scavenge ROS and protect cells from oxidative stress. This prevented the transformation of BMSCs from an osteogenic to an adipogenic phenotype, effectively promoting bone defect healing in a diabetic mouse model.^[^ ^43^ ^]^ We also utilized Wang et al.’s diabetes cell model^[^ ^29^ ^]^ to validate the function of HLBP. Similarly, HLBP enhanced the osteogenic function of hBMSC. Wang et al. encapsulated metformin in silica nanoparticles and combined this with stromal cell‐derived factor‐1 (SDF‐1) and a thermosensitive poly(*D,L*‐lactide)‐block‐poly(ethylene‐glycol)‐block‐poly(*D,L*‐lactide)‐(PDLLA‐PEG‐PDLLA) copolymer to form a hydrogel. Utilizing SDF‐1's ability to recruit MSCs and metformin's effect in reducing ROS levels, the system activates osteogenesis under high‐glucose conditions through the AMPK/β‐catenin pathway, thereby promoting periodontal bone regeneration in a T2DM environment. Recent studies with characteristics similar to those of HLBP hydrogels have been summarized in a comparative table provided in the supplementary materials (Table S2, Supporting Information).^[^ ^43^, ^44^, ^45^, ^46^, ^47^, ^48^, ^49^, ^50^, ^51^, ^52^ ^]^ In contrast to Wang^[^ ^29^ ^]^ et al.’s use of hydrogel delivery of metformin for clearing elevated levels of ROS in a hyperglycemic environment to promote osteogenesis, our chosen HLBP not only reduces ROS but also directly promotes osteoblast proliferation, offering a new option for treating diabetic bone defects. It can be seen that materials capable of effectively reducing high levels of ROS around the organization can help the faster recovery of bone defect sites. Compared to materials that directly reduce ROS levels around the tissue to promote bone defect repair, we chose a drug (LBP) that also promotes BMSC proliferation activity and osteogenic differentiation. At the same time, we also verified it in a high‐sugar simulated diabetes cell model and BKS‐db mouse bone defect model.

Currently, commonly used bone defect models mainly include the sheep iliac crest bone defect model,^[^ ^53^ ^]^ beagle vertical bone defect model, rabbit open tooth socket bone defect model, rat skull defect model,^[^ ^54^ ^]^ and mouse skull defect model.^[^ ^43^ ^]^ For ethical reasons, we require a dependable and consistent bone defect model capable of maintaining elevated blood glucose levels. Based on previous research, the genetic characteristics of BKS‐db mice make them the optimal choice for this purpose.^[^ ^55^ ^]^ Therefore, we chose the BKS‐db skull defect model as the animal model for this study. Meanwhile, we referred to Zhang et al.’s study on a diabetic bone defect mouse model^[^ ^43^ ^]^ and created a critical size bone defect of 3 mm to examine the in vivo functional performance of HLBP. In our study, the observation times we chose were postoperative second week, fourth week, and eighth week, which is consistent with the previous studies.^[^ ^34^, ^43^ ^]^ The bone defect healing area reached over 80%, proving the effectiveness of HLBP in osteogenesis in vivo. However, it is evident that complete healing remains unattainable by the eighth week, possibly due to the fact that improvement of the microenvironment is just one of several crucial factors in the bone healing process. Previous studies have shown that appropriate mechanical force is also essential for bone healing,^[^ ^56^ ^]^ and it is possible that the mouse skull defect model used in this study did not provide effective and suitable mechanical stress stimulation at the defective site to facilitate bone healing. As a result, similar to previous research findings, there was an inability to achieve 100% healing.

Our study still has limitations. HLBP was only applied in cranial defects of diabetic model mice, and the bone defect shape was regular, which can only demonstrate that the HLBP composite scaffold is suitable for repairing regular non‐load‐bearing bone defects. In the next step, irregularly shaped load‐bearing bone defect models should be established to fully demonstrate and reflect the performance of HLBP. It is also necessary to extend the time spent on animal models to evaluate the long‐term bone repair effect of HLBP. However, given the complexity of clinical diabetes‐related bone damage, it may not be sufficient for HLBP with its ROS clearance and osteogenic effects alone. Therefore, further research is needed to improve HLBP in order to transition from basic research to clinical application as soon as possible.

## 4 Conclusion

In this study, we report the development of an LBP‐loaded hydrogel (HLBP) with both physical and chemical double cross‐linked networks. The hydrogel demonstrated strong ROS scavenging activity and effectively promoted osteogenesis in diabetic bone defects. Our results reveal that LBP exerts its antioxidative effects by activating the NRF2/HO‐1 signaling pathway, reducing oxidative stress, and restoring a favorable osteogenic microenvironment. Moreover, HLBP provided a stable, bioactive scaffold that supported in situ bone regeneration. In vivo, experiments confirmed that HLBP significantly accelerated bone defect healing and enhanced new bone formation in a diabetic mouse model. These findings highlight the potential of LBP‐loaded hydrogels as a promising approach to address oxidative stress‐related challenges in diabetic bone repair. Further studies and clinical trials are needed to optimize its therapeutic application in bone tissue engineering.

## 5 Experimental Section

Polyvinyl alcohol, polyethylene glycol diacrylate (PEGDA, MN: 4000 kda), alpha‐ketoglutaric acid, and Lycium barbarum polysaccharide were all procured from Aladdin (China). The experiment used high‐purity water throughout.

### The Production Process of Hydrogel

Taking HLBP10 as an example: first, 10 g of polyvinyl alcohol (PVA) was dissolved in 50 mL of deionized water to obtain a 20% PVA solution at 95 °C, then cooled it to room temperature. Next, mixed 20 w/w % acrylic acid, 5 w/w % PEGDA, 0.2 w/w % α‐ketoglutaric acid (photoinitiator), and 10 w/w % PVA uniformly to obtain a precursor solution for photo‐curable hydrogel. Then added LBP at a mass ratio of 10 mg g^−1^ and stir evenly. Injected the precursor solution into a glass mold with a 10 mm spacer and freeze the resulting gel at −20 °C for 4 h in the refrigerator, followed by thawing at room temperature for another two hours. This freezing and thawing process were repeated three times. Finally, the gel was exposed to UV light at a wavelength of 365 nm for one hour to achieve photo‐crosslinking of HLBP hydrogel. Last, the prepared HLBP gel was freeze‐dried for later use.

According to the immersion method specified in GB/T 16886.5‐2017, the concentration of LBP in the immersion solution of HLBP0, HLBP4, HLBP6, HLBP10, and HLBP20 was ≈0, 0.8, 1.5, 2, and 4 mg mL^−1^ respectively.

### FTIR

Fourier‐transform infrared spectroscopy (FTIR) in attenuated total reflectance (ATR) mode (Bruker Optics, Germany) was used to characterize the gel structure and groups. The wavelength range was 400–4000 cm^−1^.

### SEM

The dried samples were treated with gold spray and the microscopic morphology was observed using a benchtop scanning electron microscope (Prox, The Netherlands).

### Stretching Experiment

After the gel reached swelling equilibrium, it was cut to a dumbbell shape (4 × 50 mm), and the tensile test was performed using an electronic universal testing machine (INSTRON 3366, USA) at a tensile rate of 50 mm min^−1^. Three parallel samples were set for each sample to reduce the error.

### Contact Angle

The water contact angle of the hydrogel film was measured using a water contact angle measuring instrument (DSA25E, Germany), with three parallel samples set for each sample to minimize error. Water Contact Angle Measurement: The hydrophilicity of the material was evaluated by measuring the contact angle formed between a water droplet and the material's surface. A contact angle less than 90° indicated hydrophilic properties, while an angle greater than 90° suggested hydrophobicity.

### Swelling

A certain amount of dry hydrogel was placed in a glass dish and immersed in deionized water. At different time points, the hydrogel samples were removed and the surface water was wiped with qualitative filter paper, and the mass was weighed. The equilibrium swelling rate of the hydrogel was then calculated according to the following formula.

(1) $$\mathrm{Swelling}=\left(\mathrm{Wd}-W0\right)/W0\times 100\%$$

Note: *W*_0_ is the initial mass of the hydrogel, *g*; *W*_d_ is the mass of the hydrogel immersed at different time points, *g*.

### Degradation

A certain amount of dry gel was immersed in 10 mL of PBS solution and placed in a constant temperature shaking box at 37 °C. Samples were taken every other day, carefully cleaned and totally dried, and the mass was weighed. The PBS solution was replaced every day, and three parallel samples were set for each sample to reduce errors. The degradation rate of the hydrogel was then calculated according to the following formula.

(2) Degradation=Wd/W0×100%

Note: *W*_0_ is the initial mass of the hydrogel, *g*; *w*_d_ is the mass of the hydrogel immersed at different time points, *g*.

### Gradient Strain Loading–Unloading Test

The final strain values were set at 100%, 200%, 300%, 400%, 500%, and 600%. When the specimen was stretched to the predefined strain, it was returned to its original gauge length at the same rate. This process was conducted to obtain the loading–unloading curves of the hydrogel under gradient strain conditions.

### Cyclic Loading–Unloading Test

The final strain was set at 600%, and the cycle number was set to 10. The cyclic loading–unloading stress–strain curves of the hydrogel specimen were obtained to analyze its fatigue resistance and self‐recovery performance.

### Cell Culture

Cells were derived from human‐derived bone marrow mesenchymal stem cells (HUXMA‐01001, Cyagen, China) and human umbilical vein endothelial cells (HUVEC‐20001, Cyagen, China) purchased by Cyagen. The cell culture medium contained cell elevated glucose basal medium (88%) (Gibco, USA), fetal bovine serum (10%) (Gibco, USA), and penicillin‐streptomycin (1%) (Gibco, USA). Cells were cultured in an incubator at 37 °C with 5% CO_2_ and 95% relative humidity. The medium was changed every three days. When the confluence degree reached 80%, hBMSCs and HUVEC were trypsinized and passaged. In the subsequent co‐culture experiments with the material, the cells were placed in the medium containing the material extract for the experiments.

### Intracellular ROS Measurement

Cells were incubated with 5 µm of H2DCFDA at 37 °C for 30 min and then washed with PBS. Intracellular ROS level was measured by a fluorescence microscope (LSM980; ZEISS, Germany). All images were captured under the same conditions, and the fluorescence was quantified by ZEN software (ZEISS, Germany).

### Cell Counting Kit‐8 (CCK‐8) Assay

The hBMSCs cells were cultured with different groups of scaffolds after 0, 3, and 7 days, and evaluated the cytotoxicity and cell proliferation using a cell Counting Kit 8 (Abbkine, Cat# KTA1020). Temporarily, at 3 h after adding the CCK‐8 reagent to 10% of the medium, the absorbance (450 nm) was assessed utilizing a microplate reader (Bio‐TekELx800) and proliferation curves for cells of various groups were then plotted.

### Live/Dead Assay

For a visual assessment of cell culture with different groups of scaffolds, a live/dead kit (Baiaolaibo, China, Cat#HR8279) was employed to assess cell viability after culturing with scaffolds for 3 days. Dye diluent and serum‐free culture medium were used to dilute calcein‐AM and EthD‐I to prepare a live/dead solution, and 500 µL of the solution was added. Following that, cells were incubated for 15 min. Live and dead cell images were acquired using a fluorescence microscope (Nikon, Tokyo, Japan). Live cells were green‐stained with calcein‐AM (CL), while dead cells were red‐stained with Propidium iodide (PI).

### Scratch Assay

The hBMSCs cells were cultured with different scaffolds until they reached 100% confluence. Straight lines were then scratched through the middle of the cells in every well. The full medium was substituted with serum‐free medium. Images of scratches were captured after 0, 12, and 24 h using the microscope. The average scratch width was quantified using ImageJ 6.4 software, and the rates of scratch wound healing were calculated.

### Transwell Migration Assays

Transwell migration was used to determine cell metastasis capacities. Temporarily, 6–10 × 10^4^ cells were placed into the upper chamber with an 8 µm pore size (354576, Corning, New York, USA) with 200 µL of serum‐free medium, while 600 µL of supplemented with 10% FBS was added to the lower chamber. Following the transfection period of 24 h, the cells in the upper chamber were removed using a cotton swab, and the cells in the lower chamber were then fixed and stained. Finally, the number of migrated cells was quantified and imaged under a light microscope.

### In Vitro Angiogenesis Characterization

To detect tube formation, plates were prepared with matrigel at 4 °C, and 200 µL of HUVEC cells were seeded into 48‐well plates at a concentration of 3 × 10^4^ and cultured using the corresponding medium. After 12 h of culture, HUVECs were photographed using a microscope to count the number of tubes.

### In Vitro Osteoblast Assay

The hBMSCs were seeded in a medium with or without material extract. When the degree of cell fusion reached 70%, the OriCell hBMSCs Cell Osteogenic Differentiation kit was used. The differentiation medium was changed every 3 days.

### ALP Staining

After 7 days of co‐culture and induction, cells were fixed with 4% PFA for 30 min, and ALP activity was measured by an ALP staining kit. After incubation with ALP chromogen for 30 min, the results were observed under a microscope.

### Alizarin Red Staining (ARS)

The calcium nodules generated by the cells were stained with the ARS kit. After 21 days of co‐culture, cells were washed with PBS, fixed with 4% PFA for 30 min, stained with ARS for 15 min, and observed for calcium nodules by camera and microscope.

The ARS kit was used to stain calcium nodules formed by cells. After co‐culturing hBMSC cells and HLBP10 for 21 days, the cells were washed with PBS, fixed in 4% PFA for 30 min, stained with ARS for 15 min, and then observed under a camera and microscope for calcium nodules. Dissolved calcium nodules using CPC (Cetylpyridinium Chloride), collected the solution and then performed quantitative analysis using a microplate reader (Gen5; BioTek Instruments).

### Alcian Blue Staining (AB)

A chondrogenic differentiation kit (Cyagen, Santa Clara, CA, USA) was utilized to induce chondrogenic differentiation of BMSCs. Shortly, BMSCs at the third passage were plated into 24‐well plates (1 × 10^5^ cells well^−1^) and incubated with the chondrogenic medium. After incubation for 0, 7, and 14 days, BMSCs were fixed with 4% paraformaldehyde (PFA) for 15 min and rinsed with phosphate buffer saline followed by incubation for 40 min with 0.1% Alcian blue 8GX (Solarbio, Beijing, China). The optical density at 590 nm was detected.

### Oil Red O Staining (ORO)

The hBMSCs were seeded in a medium with or without material extract. When the degree of cell fusion reached 70%, the OriCell hBMSCs cell adipogenic differentiation kit was used. The differentiation medium was changed every 3 days. On day 21, Oil red O staining was conducted. In brief, after flushing with phosphate‐buffered saline (PBS), cells were fixed with 10% formalin. The cells were then rinsed with 60% isopropyl alcohol for 10 min and incubated with 0.3% Oil red O. After 100% isopropyl alcohol was added to the stained cells, quantitative measurement was conducted by determining their spectrophotometric absorbance at 520 nm.

### Bodipy 493/503 Staining

In the same manner as the cultivate described above, after 21 days, staining was performed using the Bodipy staining kit. After staining, the fluorescence intensity was measured under a fluorescence microscope.

### Immunofluorescence Staining (IF)

Cells were fixed with 4% PFA for 30 min and then permeabilized with 0.2% Triton‐100 in PBS for 20 min. Then, the BSA (5% w/v) was used for blocking at room temperature for 1 h. After the blocking, cells were incubated with primary antibody against F‐Actin, OCN, RUNX_2_, Pparγ, or SOX9 at 4 °C overnight and further stained with a secondary antibody with green or red fluorescence respectively at room temperature for 1 h. Finally, DAPI was used to stain the nuclei. The results were observed under a fluorescence microscope (LSM980; ZEISS, Germany).

### In Vitro ROS Scavenging of Hydrogels

The ROS scavenging activity of hydrogels was evaluated in vitro using the DPPH assay. Hydrogel samples HLBP0 and HLBP10 were treated with 100 µm DPPH solution and incubated in a 24‐well plate on an oscillator at 37 °C for 24 h. The antioxidant drug TEMPOL (1 mg mL^−1^) was used as a positive control. The absorbance of the incubation solution was measured at 517 nm using a microplate reader (CYTATION 5, BioTek, USA). The ability of the hydrogels to scavenge DPPH free radicals was determined. The percentage of ROS inhibition (%) was calculated as (ADPPHA_sample)/ADPPH × 100, where ADPPH was the absorbance of the DPPH solution and A_sample was the absorbance of the sample solution.

### HUVEC Cell Angiogenesis Experiment

After processing HUVEC cells (OriCell, HUVEC‐20001) under different conditions, they were digested with trypsin and resuspended in a complete culture medium. The cell count was performed, and 1 × 10^4^ cells were seeded into a 96‐well plate pre‐coated with Matrigel (Corning Matrigel, USA). After incubation in a cell culture incubator for 4 h, the formation of tube‐like structures was observed under a microscope. If no structures were observed, the incubation time could be appropriately extended. The culture medium was then removed, and diluted calcein‐AM was added followed by an incubation period of 5–10 min. Fluorescence microscopy was used to observe and photograph the cells.

### Mice Skull Defect Model and Implantation of Materials

All procedures followed the Chinese national guidelines for the care and use of laboratory animals (LunShen (Yan) 2024‐527‐1). BKS‐db mice aged 8 weeks were obtained from the Chengdu Yaokang Biotechnology Co. LTD. Before and after the operation, they were maintained in a specific pathogen‐free (SPF) environment with freely available water and standard mouse chow. All surgical procedures and cooperative handling were conducted in accordance with protocols approved by the Ethics Committee of the Sichuan Academy of Medical Sciences Sichuan Provincial People's Hospital.

All surgeries were conducted by senior veterinarians with extensive experience. Before surgery, mice were fed for 1 week in separate cages to adapt to the environment. Anesthesia was performed by inhalation of isoflurane. After incising the skin on the top of the head, a circular full‐thickness bone defect with a diameter of 3 mm was created by a drill. Then the materials were physically cut into appropriate sizes, filled into the defect areas of mice in different groups, and the wounds were sutured layer by layer. In the Control group, after drilling, no material was filled, and the skin incision was simply sutured closed. In the Sham group, only a skin incision of the same length was made on the top of the head without drilling into the skull, and then the wound was sutured directly. During the initial three postoperative days, each mouse was administered analgesic therapy via subcutaneous injection of meloxicam (meloxicam injection, SL001305, China) at a dosage of 0.02 mL 10 g^−1^ day^−1^.

### Blood Glucose Measurement

Blood glucose was measured weekly throughout the mouse cycle, blood glucose monitoring was carried out by a dedicated blood glucose meter (VGM30‐431, China).

### Detection of In Vivo ROS Expression in Skull Defect Area

After materials implantation, the mice were anesthetized by 1% pentobarbital sodium and 100 µm DCFH‐DA was injected into the defect sites on days 3, 7, and 14. ROS levels in each group were monitored using an IVIS Spectrum (IVIS SPECTRUM, PerkinElmer, USA). Fluorescence intensity was used to determine the ROS scavenging capacity of the materials.

### Analysis of Microcomputed Tomography

The harvested calvariae were scanned using micro‐CT (40 kV, 110 mA, no filter). 3D reconstruction images were generated.

### Histological Assessment

Harvested calvariae were decalcified in 10% EDTA for 4 weeks. The decalcified calvarias were embedded in paraffin and sliced into sections with a thickness of 5 µm. These sections were then used for H&E and Masson's trichrome staining according to the manufacturer's instructions. Immunohistochemical (IHC) staining of Runx_2_, SOX9, Pparγ, OCN, and Trap was performed to analyze. Cell nuclei were counterstained with haematoxylin (Solarbio). The stained slides were imaged using a slider scanner (Axioscan 7, ZEISS, Germany).

### Immunofluorescence Staining (IF)

Tissues were fixed in 4% paraformaldehyde for 48 h and incubated in 15% diethyl pyrocarbonate–EDTA (pH 7.8) for decalcification. Then, specimens were embedded in paraffin and sectioned at 5 µm. Immunohistochemistry was performed using a trichostatin biotin amplification system (PerkinElmer Life Sciences) according to the manufacturer's instructions using antibodies against RUNX_2_, OCN, Pparγ, Ctsk, and SOX9. Frozen bone samples were thawed at room temperature for 10 min, rehydrated with PBS, and blocked for 1 h with 10% horse serum and 0.2% Triton X‐100 in PBS, followed by primary antibodies incubation overnight at 4 °C. Then, samples were washed three times with PBS and incubated by secondary antibodies for 1 h followed by washing three times with PBS. The samples were mounted with a fluoroshield mounting medium with 4′,6‐diamidino‐2‐phenylindole (DAPI; Abcam, ab104139).

### Western Blotting (WB)

Treated cells were isolated using radioimmunoprecipitation assay (RIPA) lysis buffer (P0013C, Beyotime, Shanghai, China) with protease and phosphatase inhibitors (P1045, Beyotime, Shanghai, China) on ice and centrifuged at 12 000 g for 10 min at 4 °C, and the supernatants were collected. Then BCA protein assay measured the concentrations of the lysates. Proteins were separated using 8% or 12% SDS‒PAGE and then transferred to polyvinylidene fluoride (PVDF) membranes (1620177, Bio‐Rad, CA, USA). The membranes were blocked with Tris‐buffered saline containing Tween‐20 (TBST) containing 5% nonfat dry milk (D8340, Solarbio, Beijing, China) for 1 h at room temperature and then incubated with primary antibodies overnight at 4 °C. On the second day, the membranes were washed three times with TBST for 15 min totally and incubated with species‐matched secondary antibodies (ab150077, Abcam, Cambridge, UK, 1:10000, 0.2 µg mL^−1^) at room temperature for 1 h. The protein bands were visualized by Millipore's enhanced chemiluminescence reagent. All Western blot experiments were conducted in triplicate, and the membrane images were captured using a Tanon image analysis system (5200, Tanon, Shanghai, China) and analyzed with Image J software (version: 1.52n, author: Wayne Rasband). The antibodies utilized in this experiment encompass Anti‐RUNX_2_ (1: 1000, SD208‐0) were obtained from HUABIO. Anti‐OCN (1: 1000, bs‐4917R) were obtained from BIOSS. Anti‐Pparγ (1: 1000, sc‐7273) were obtained from Santa. Anti‐SOX9 (1: 1000, 67439‐1‐Ig) were obtained from Proteintech. Anti‐TRAP (1: 1000, sc‐376875) was obtained from Santa. Anti‐CTSK (1: 1000, sc‐48353) were obtained from Santa.

### Statistical Analysis

Analyses were performed using SPSS software for Windows version 22.0. All experiment animals were euthanized at different time points. Student *t*‐tests and one‐way analysis of variance (ANOVA) and with Levene's test for homogeneity of variance, followed by Tukey's post hoc test or Bonferroni post hoc test based on the various comparisons to be made and the statistical indication of each test, were used. After the normal test, two‐way repeated measurement ANOVA with the Bonferroni post hoc test was used to assess tests from the same animals over multiple time points. *P*‐values were recorded in the statistical chart. Sample sizes were incorporated into the text and figure legends, and individual data points from each replicate were depicted as small circles in all figures.

### Ethics Committee Statement

Institution: Medical Ethics Committee, Sichuan Academy of Medical Sciences, and Sichuan Provincial People's Hospital

Results of review: Agreed to carry out the experiment in accordance with the approved experimental protocol.

## Conflict of Interest

The authors declare no conflict of interest.

## Author Contributions

W.Z. dealt with writing the review and editing, writing the original draft, visualization, methodology, investigation, data curation, and conceptualization. W.L. dealt with methodology, investigation, and data curation. L.X. dealt with methodology and investigation. N.H. dealt with the investigation. K.X. dealt with the investigation. C.L. dealt with the investigation. F.W. dealt with writing the review and editing, and investigation. W.Z. dealt with the investigation. J.H. dealt with writing the review and editing and funding acquisition. H.C. dealt with writing the review and editing, supervision, project administration, funding acquisition, and conceptualization.

## Supporting information



Supporting Information

## Data Availability

The data that support the findings of this study are available in the supplementary material of this article.
