# Supplementary material for: Lycium‐Barbarum Polysaccharide‐Loaded Dual‐Crosslinked Rigid Hydrogel Enhances Bone Healing in Diabetic Bone Defects by Scavenging Reactive Oxygen Species
Source: Adv Healthc Mater. 2025 Mar 17;14(11):2404741. doi: 10.1002/adhm.202404741 (PMC12023829; doi:10.1002/adhm.202404741)
Supplement: Supplementary file 1 — Supporting Information [file ADHM-14-0-s001.docx]

**Supplemental Materials**

**Supplemental table**

Table S1. Tensile performance of HLBP composite stent.

Table S2. Comparison of HLBP Hydrogel with Recent Studies.

**Supplemental figures**

Supplementary Figure 1. The overall perspective of HLBP.

Supplementary Figure 2. The SEM images of HLBP.

Supplementary Figure 3. The FTIR of the HLBP.

Supplementary Figure 4. The water contact angle of the HLBP.

Supplementary Figure 5. The swelling rate of the HLBP.

Supplementary Figure 6. The tensile testing of the HLBP.

Supplementary Figure 7. Gradient Strain Loading-Unloading Test.

Supplementary Figure 8. Cyclic Loading-Unloading Test.

Supplementary Figure 9. The degradation rate of the HLBP.

Supplementary Figure 10. HLBP10 hydrogel inhibited oxidative stress and enhanced antioxidant capacity.

Supplementary Figure 11. Standard curve of LBP concentration.

**Supplemental Methods**

Measurement of Extract Solution Concentration.

**Table S1. Tensile performance of HLBP composite stent.**

**Table S1** Tensile performance of HLBP composite stent

| **Sample** | **Tensile strength (MPa)** | **Elongation at break (%)** |
| --- | --- | --- |
| HLBP 0 | 0.34 ± 0.03 | 781.0 ± 57.6 |
| HLBP 4 | 0.36 ± 0.03 | 713.9 ± 51.5 |
| HLBP 6 | 0.32 ± 0.01 | 734.2 ± 27.8 |
| HLBP 10 | 0.32 ± 0.03 | 682.3 ± 16.7 |
| HLBP 20 | 0.24 ± 0.03 | 691.8 ± 34.9 |

Note: The tensile properties of composite scaffolds loaded with different quality scores of LBP were evaluated. The numbers after HLBP represent hydrogels loaded with different mass fractions of LBP. Statistical analysis was conducted based on n = 3 per group. Data are shown as mean ± SD.

**Table S2** **Comparison of HLBP Hydrogel with Recent Studies.**

Table S2 Comparison of HLBP Hydrogel with Recent Studies

| **Author** | **CS** | **CM** | **Bio** | **AP** | **OP** | **MA** | **AM** | **PA** |
| --- | --- | --- | --- | --- | --- | --- | --- | --- |
| Zhang | Thioketal, Norbornene | UV crosslinking | High | High | High | NR | db/db mice | NR |
| Wu | BP, ASiPc | VDW | High | NR | High | NR | SD rat | Wnt |
| Yan | Ca²⁺, Sodium Alginate | Ionic Bond, CB | High | High | NR | High | SD rat | NR |
| Xie | Mel, HA-CHO | Schiff Base | High | High | High | NR | SD rat | Wnt/𝛽-catenin |
| Yang | Baicalin, HEA | Schiff Base | High | NR | NR | NR | C57 Mice | NR |
| Kim | Fibrinogen, PEO | Amide bond, PB | High | NR | NR | High | SD rat | NR |
| Maji | Col I, PSE | CFN | High | NR | High | NF | BALB/c nude | NR |
| Xu | GHA, Exo-mimics | CCL, DPC | High | NR | High | NR | Mice | Wnt/β-catenin |
| Xing | Rutin, PVA | Schiff, Boronate Ester | High | High | High | NR | SD rat | NR |
| Huang | GelMA, Mg²⁺ | MCC, Schiff | High | NR | High | NR | SD rat | NR |
| Zhong | LBP, PEGDA | Hydrogen Bonding | High | High | High | High | db/db mice | Nrf2/HO-1 |

Note: 1. CS: Composition & Structure; 2. CM: Crosslinking Method; 3. Bio: Biocompatibility;

4. AP: Antioxidant Properties; 5. OP: Osteogenic Performance; 6.MA: Manifestations of angiogenesis; 7. AM: Animal Model Validation; 8. PA: Pathway. 10. BP: Black Phosphorus; 11. ASiPc: Amino-silicon phthalocyanine; 12. VDW: van der Waals; 13. CB: Covalent bond; 14. Mel: Melatonin; 15. HEA: 2-Hydroxyethyl Acrylate; 16. PB: Peptide bond; 17. PSE: Phaseshift emulsion; 18. CFN: Collagen Fibrillar Network; 19. GHA: guanidinylated hyaluronic acid; 20. CCL: Chemical cross linking; 21. DPC: Dynamic physical cross-linking; 22. GelMA: Gelatin methacrylamide; 23. MCC: Metal Coordination Complexation; 24.Wnt: Wnt signaling; 25. NR: Not reported; 26. NF: No facilitation.

**Supplementary Figure 1. The overall perspective of HLBP.**


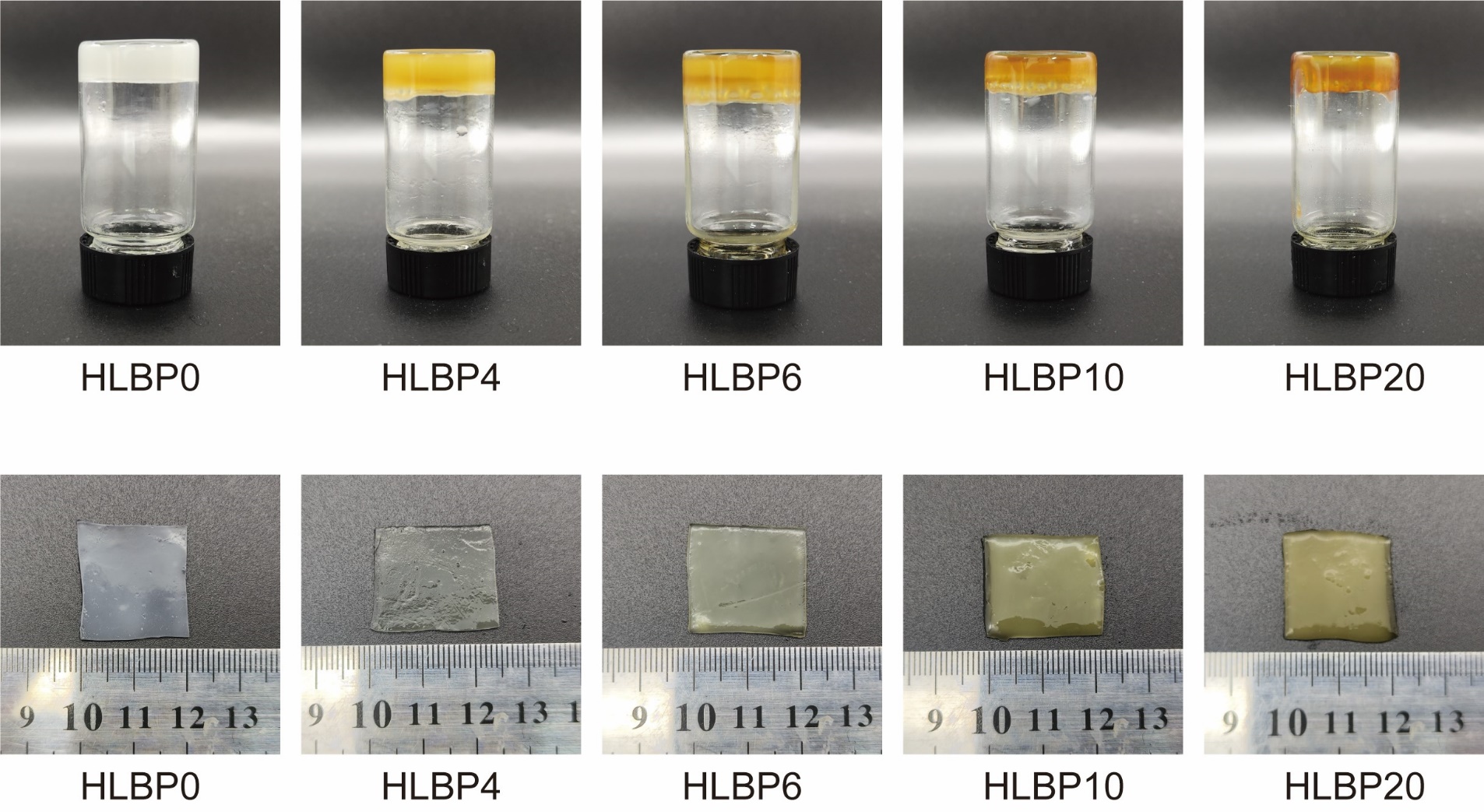


**Fig.S1** The overall appearance of the HLBP with varying grades of Lycium arbarum. The numbers after HLBP represent hydrogels loaded with different mass fractions of LBP.

**Supplementary Figure 2. The SEM images of HLBP.**


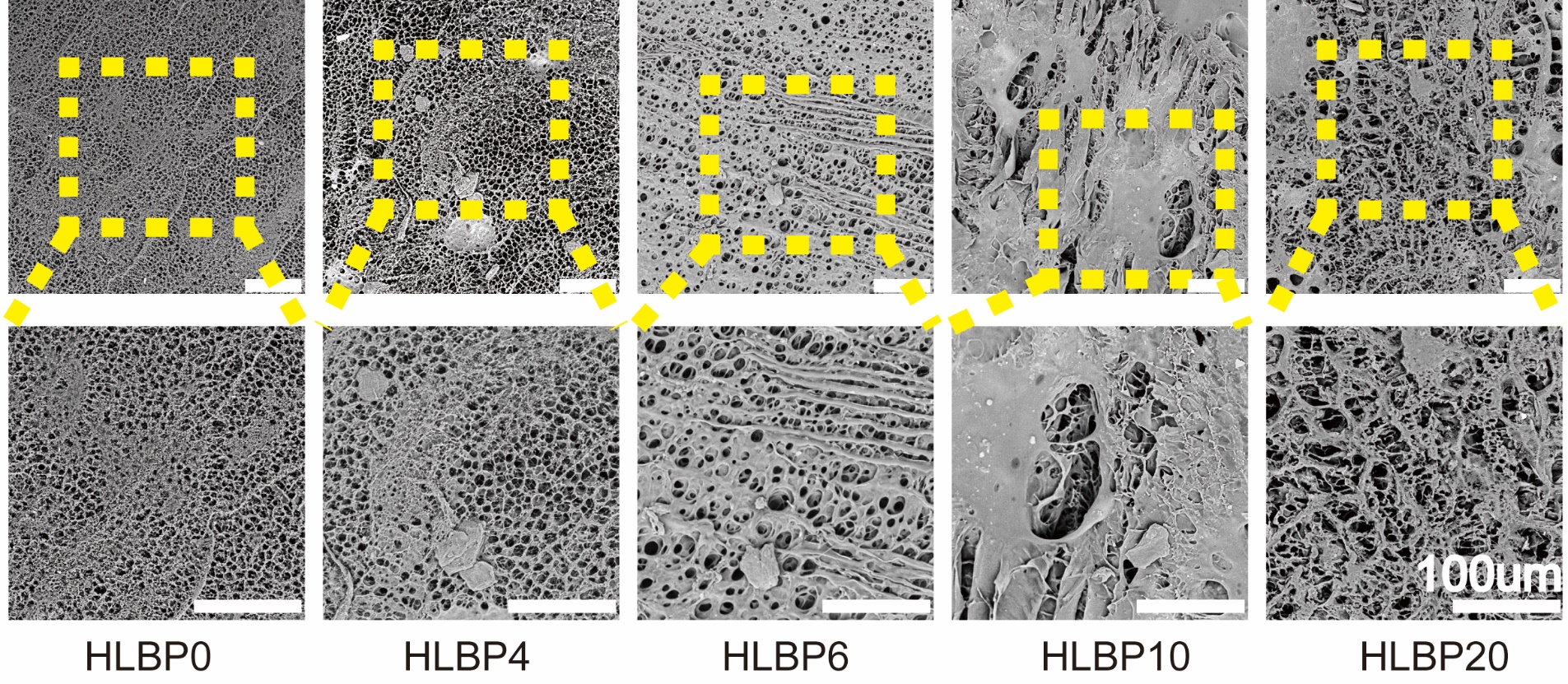


**Fig.S2** Utilize a scanning electron microscope (HITACHI Regulus 8100, Japan) for the scanning and observation of the HLBP. The numbers after HLBP represent hydrogels loaded with different mass fractions of LBP.


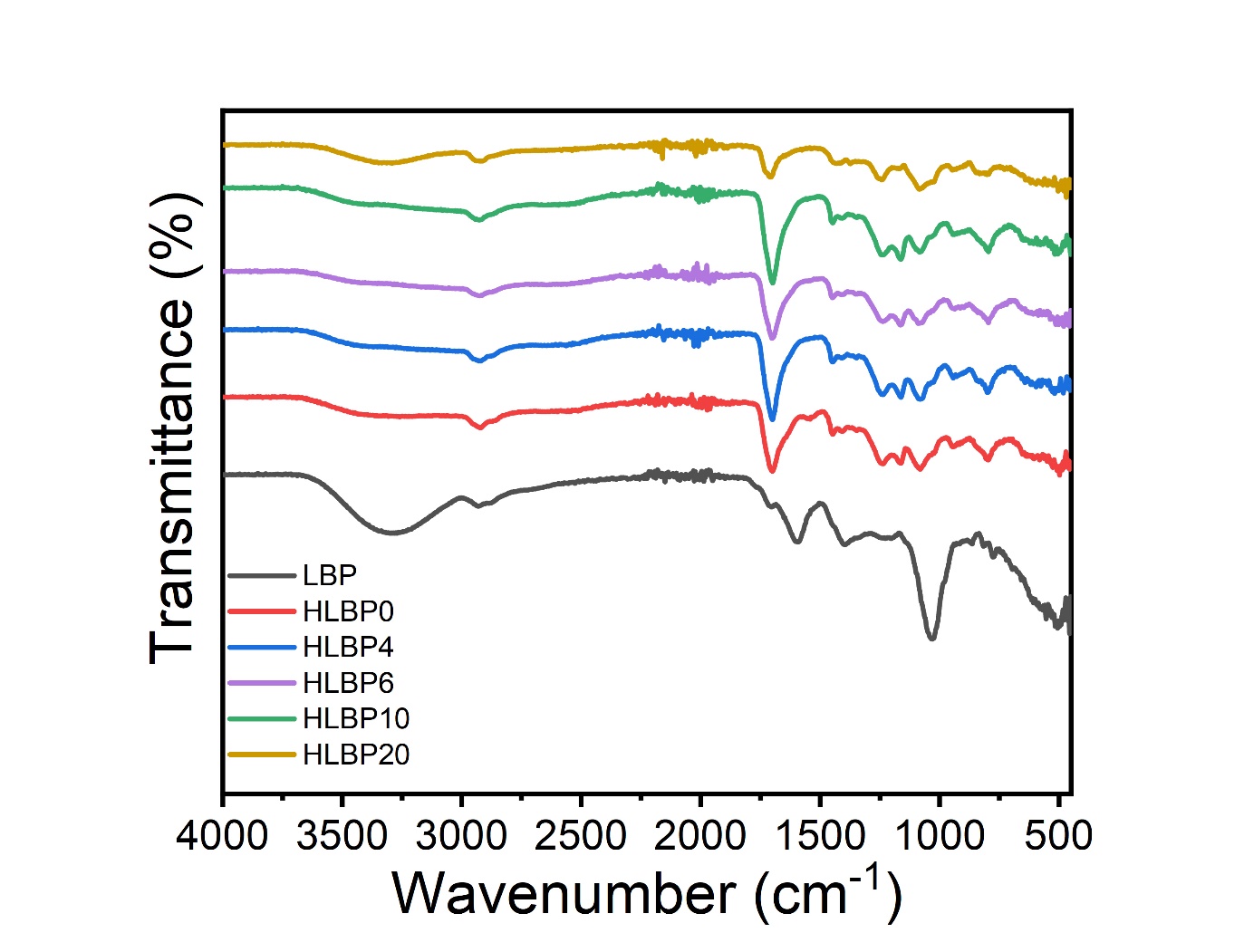
**Supplementary Figure 3. The FTIR of the HLBP.**

**Fig.S3** FTIR: Gel structure and functional groups are characterized using the attenuated total reflection mode of Fourier transform infrared spectroscopy. The wavelength range is 400-4000 cm^-1^. The numbers after HLBP represent hydrogels loaded with different mass fractions of LBP.

**Supplementary Figure 4. The water contact angle of the HLBP.**


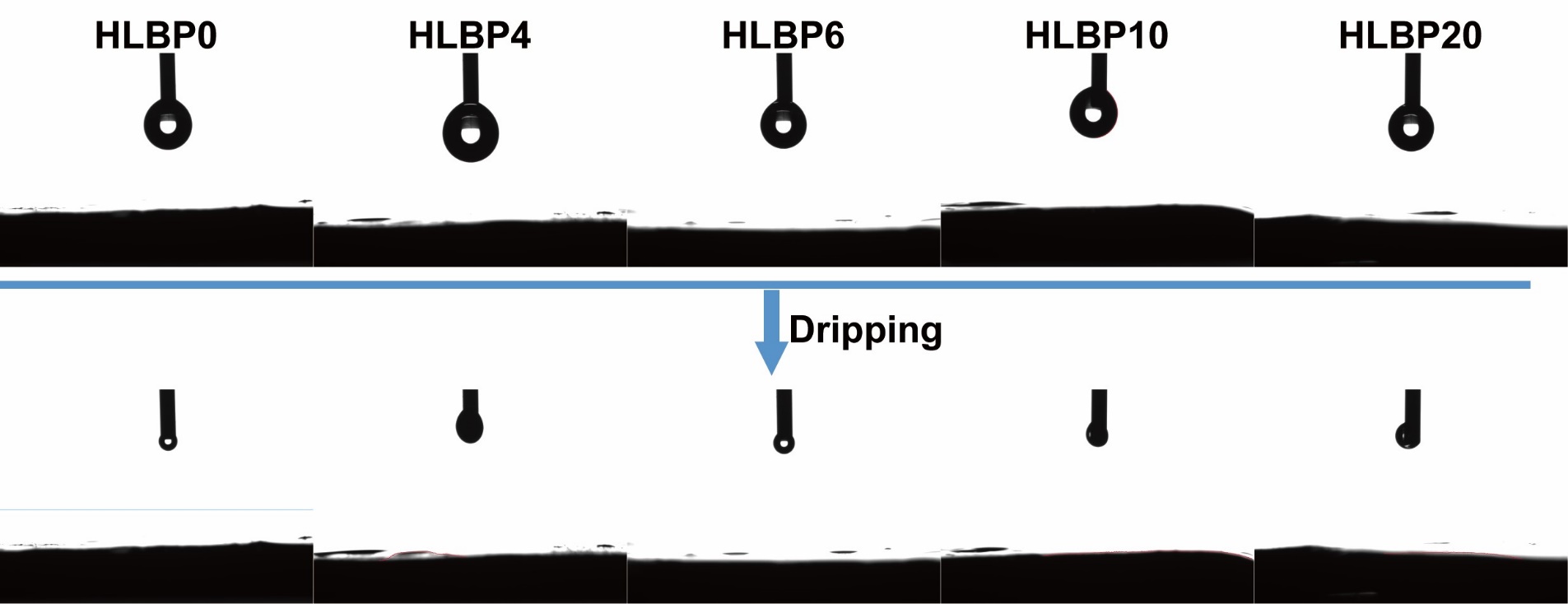


**Fig.S4** The water contact angle of the HLBP was evaluated using a water contact angle measuring instrument (DSA25E, Germany). The numbers after HLBP represent hydrogels loaded with different mass fractions of LBP.


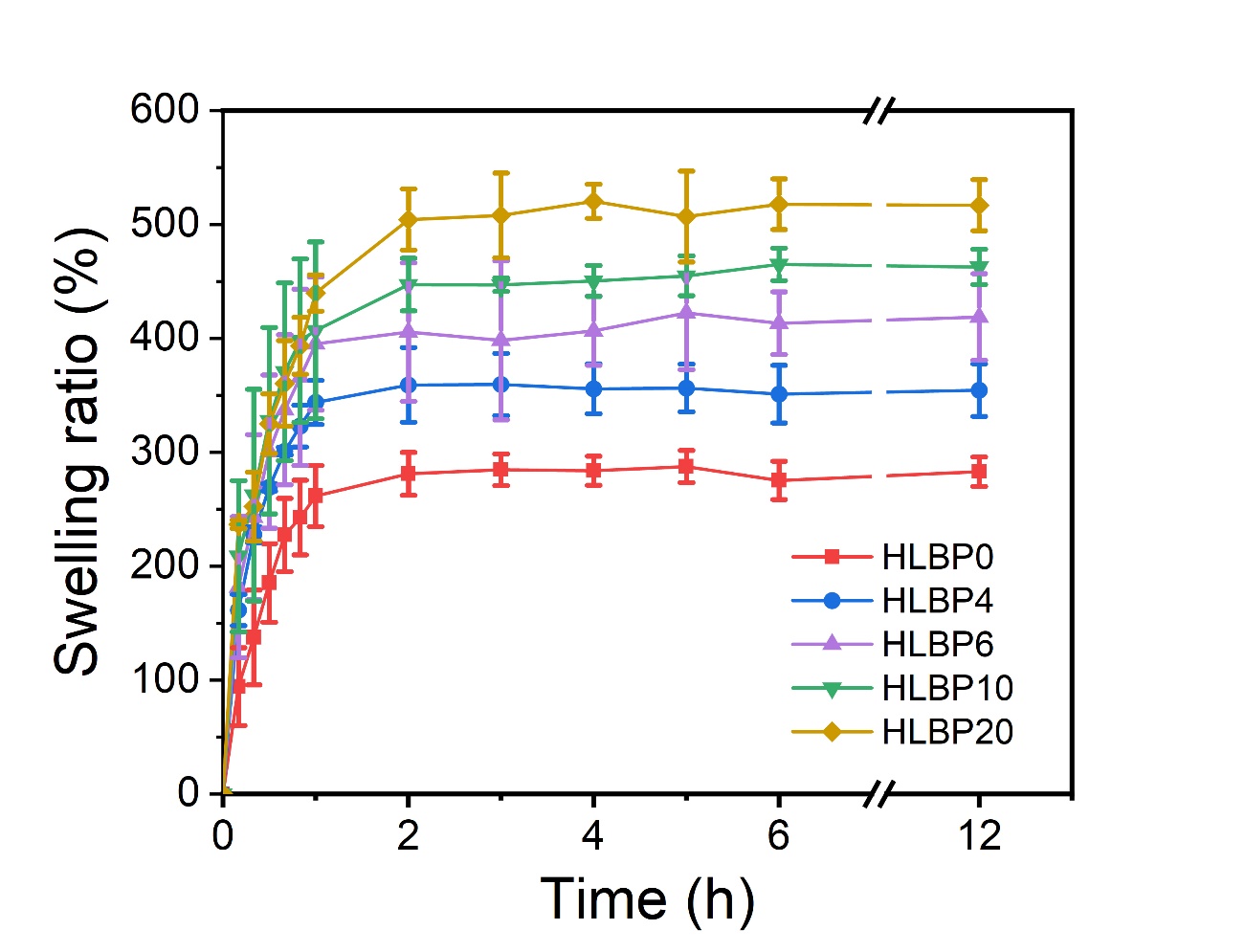
**Supplementary Figure 5. The swelling rate of the HLBP.**

**Fig.S5** The swelling rate of the HLBP in 37℃ PBS, where the swelling rate is calculated as (Wd-W0)/W0×100%, with W0 being the initial mass of the hydrogel in grams and Wd being the mass of the hydrogel at different time points after soaking. The numbers after HLBP represent hydrogels loaded with different mass fractions of LBP. Statistical analysis was conducted based on n = 3 per group. Data are shown as mean ± SD.


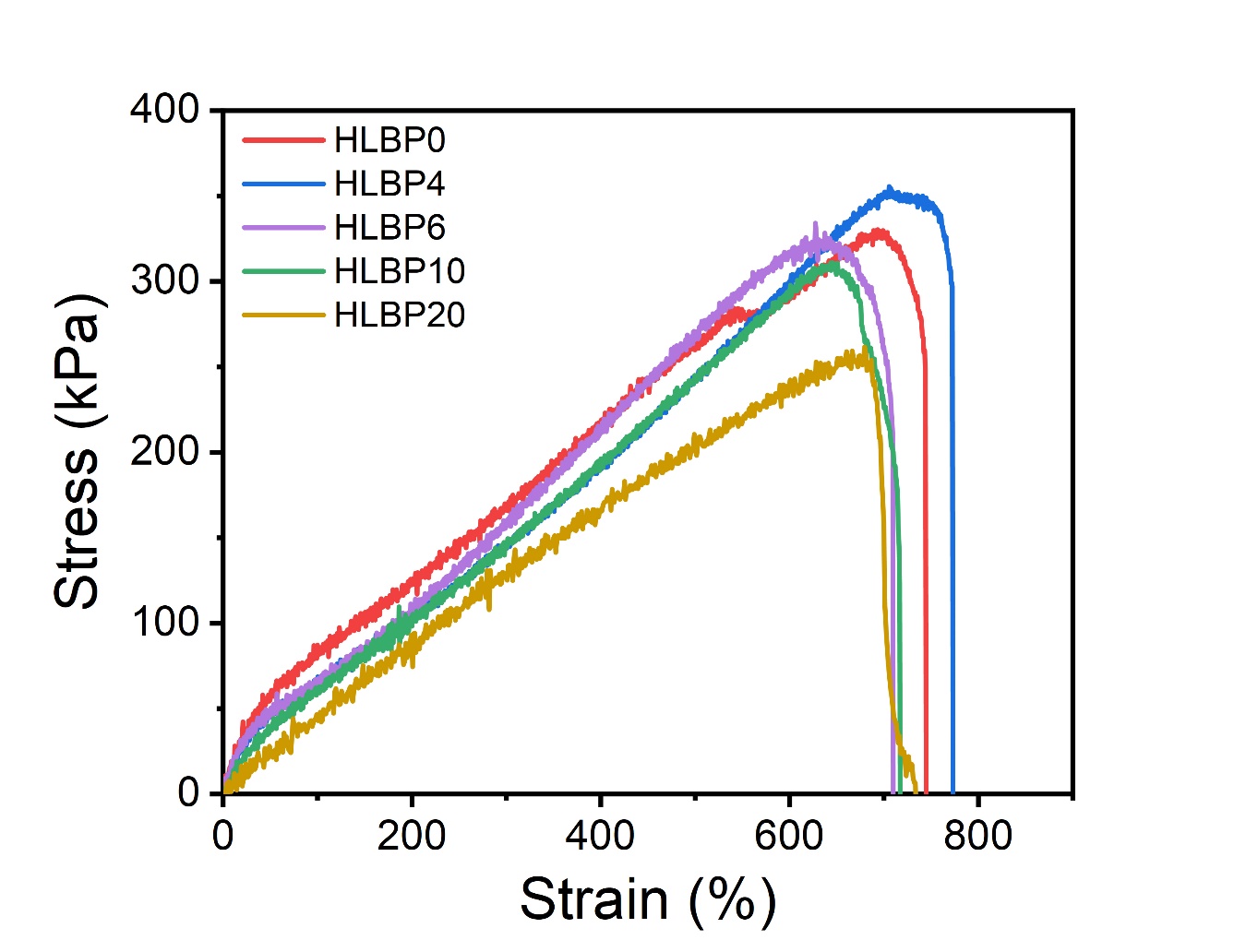
**Supplementary Figure 6. The tensile testing of the HLBP.**

**Fig.S6** Using the electronic universal testing machine (INSTRON 3366, USA) for tensile testing with a stretching speed of 50 mm/min. The numbers after HLBP represent hydrogels loaded with different mass fractions of LBP.

**Supplementary Figure 7. Gradient Strain Loading-Unloading Test.**


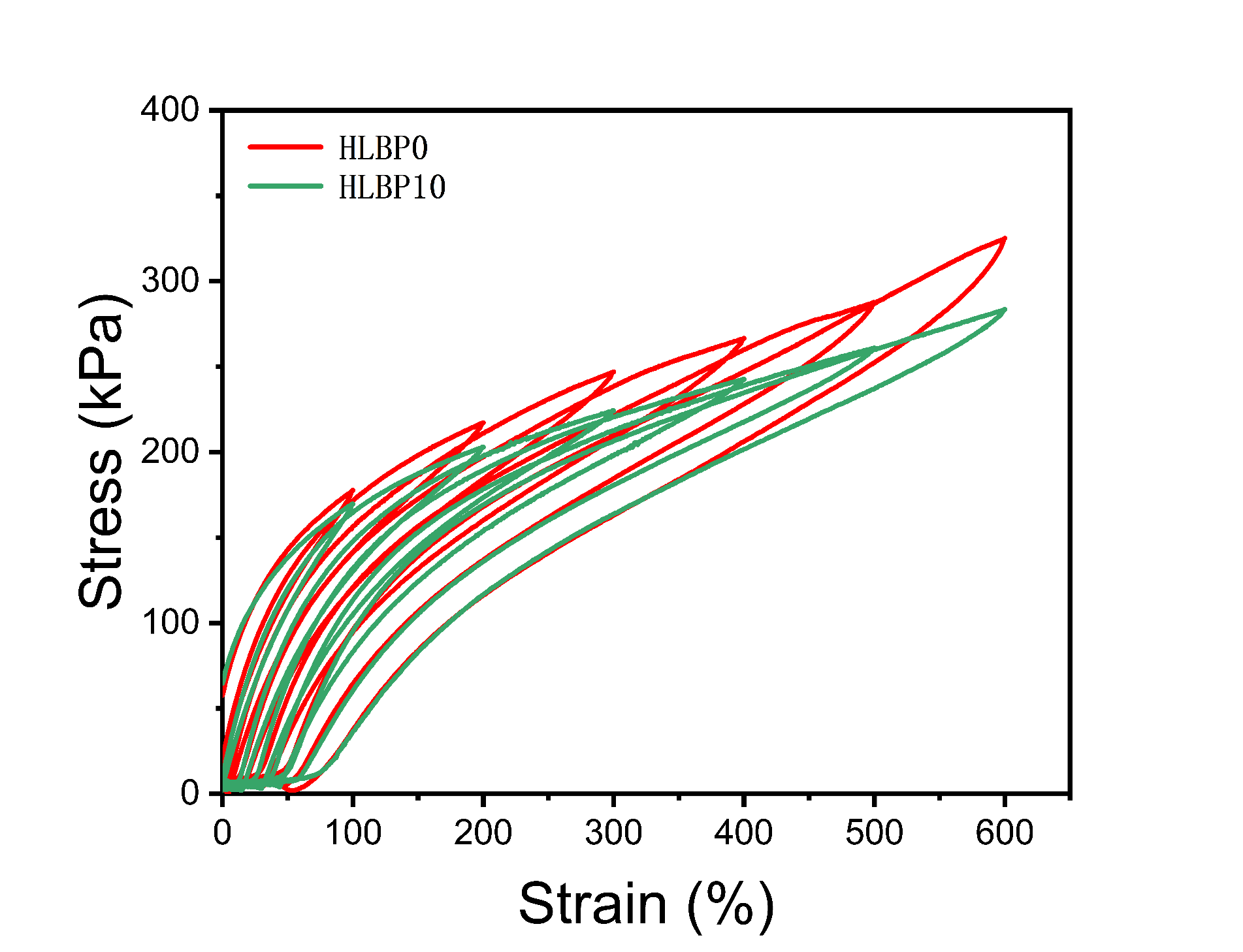


**Fig S7.** Gradient Strain Loading-Unloading Test. The final strain values were set at 100%, 200%, 300%, 400%, 500%, and 600%. When the specimen was stretched to the predefined strain, it was returned to its original gauge length at the same rate. This process was conducted to obtain the loading-unloading curves of the hydrogel under gradient strain conditions.

**Supplementary Figure 8. Cyclic Loading-Unloading Test.**


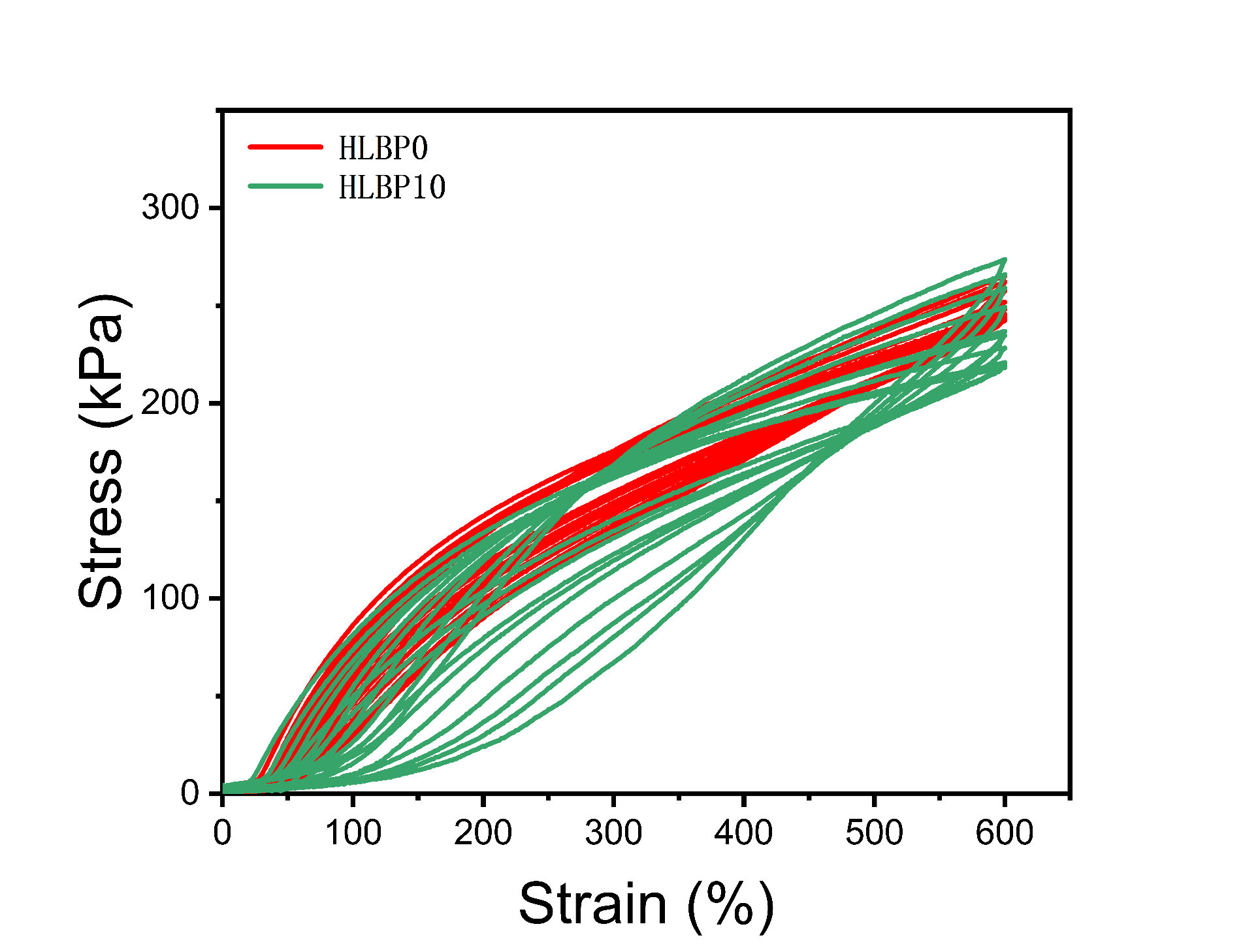


**Fig S8.** Cyclic Loading-Unloading Test. The final strain was set at 600%, with a total of 10 loading-unloading cycles. The cyclic stress-strain curves of the hydrogel specimen were obtained to analyze its fatigue resistance and self-recovery performance.

**Supplementary Figure 9. The degradation rate of the HLBP.**


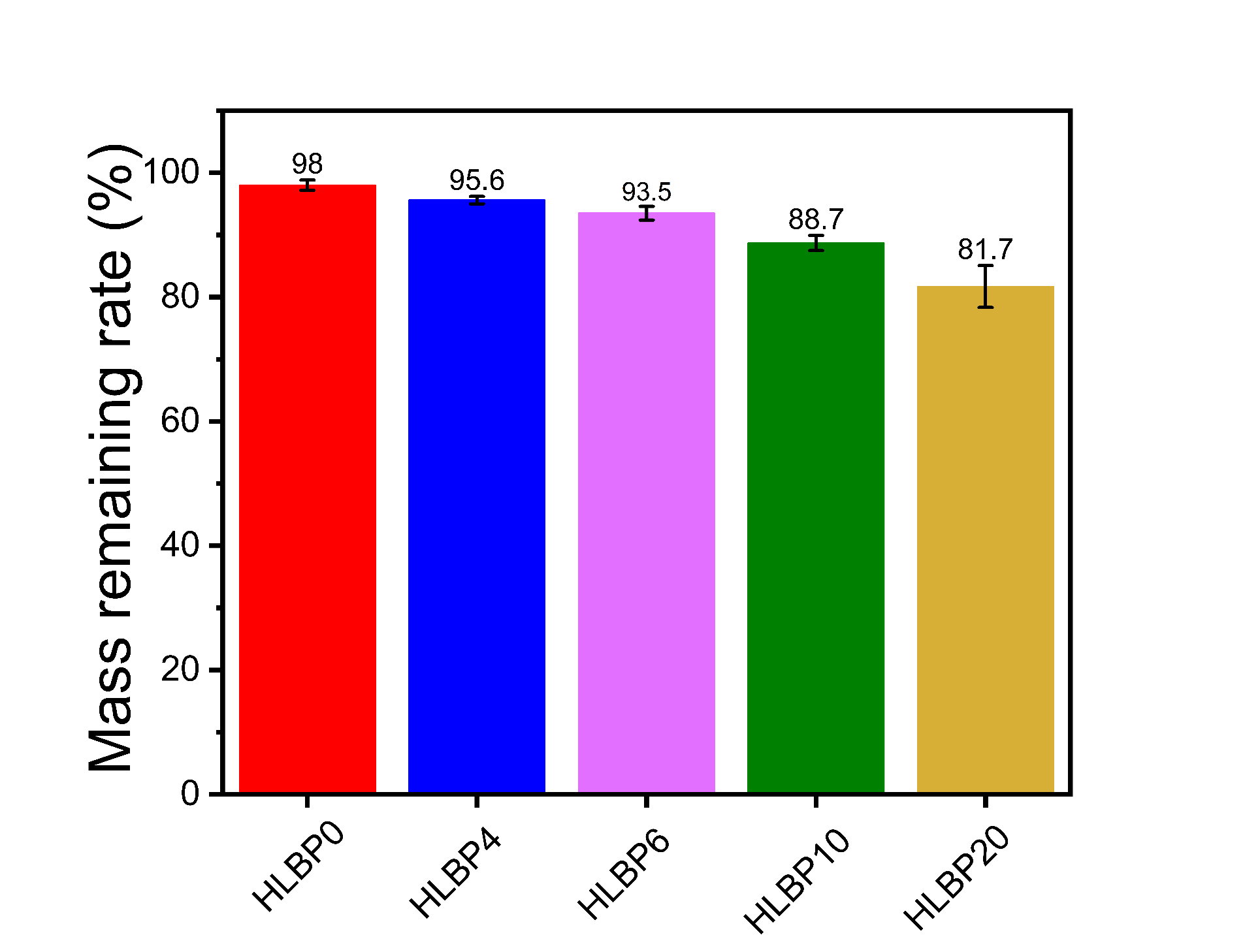


**Fig.S9** The degradation rate of the composite stent in PBS. Statistical analysis was conducted based on n = 3 per group. Data are shown as mean ± SD.

**Supplementary Figure 10. HLBP10 hydrogel inhibited oxidative stress and enhanced antioxidant capacity.
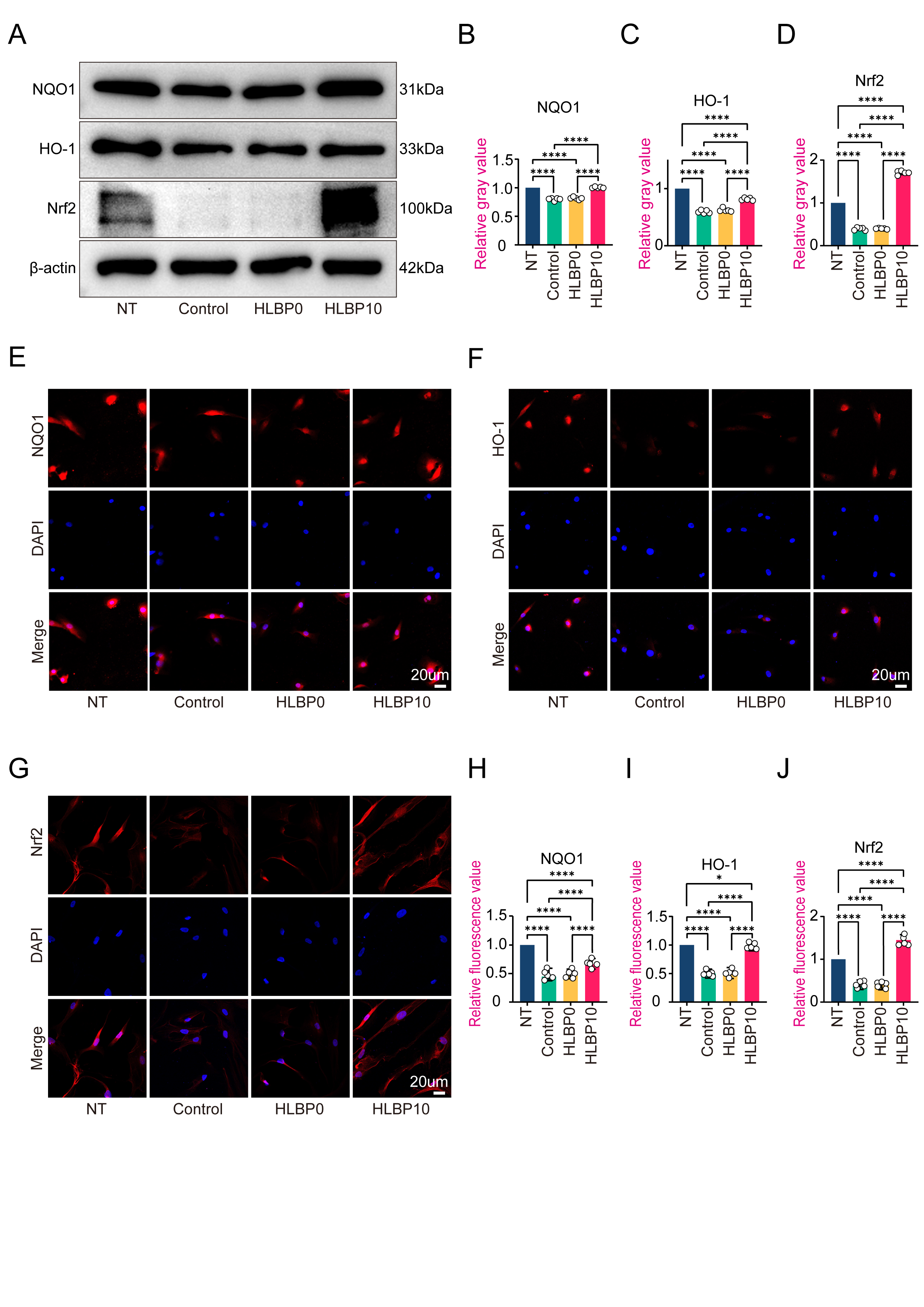
Fig.S10** HLBP10 hydrogel inhibited oxidative stress and enhanced antioxidant capacity. (A) The results of the WB (NQO1, HO-1, Nrf2) experiment for each group under different culture conditions. (B-D) Quantitative analysis of NQO1, HO-1, and Nrf2 WB results. (E)Immunofluorescence staining of NQO1 antibody protein in different groups. (F) Immunofluorescence staining of HO-1 antibody protein in different groups. (G) Immunofluorescence staining of Nrf2 antibody protein in different groups. (H-J) Quantitative analysis of NQO1 (HO-1, Nrf2) antibody protein cell immune fluorescence staining results. Data are presented as mean ± SD, n = 5, * *P* <0.05, ** *P* < 0 .01, *** *P* < 0 .001. (NT group was cultured in regular medium, Control group was cultured in high-sugar medium, HLBP0 group was cultured with HLBP extract at a mass fraction of 0, and HLBP10 group was cultured with HLBP extract at a mass fraction of 10).

**Supplementary Figure 11. Standard curve of LBP concentration.**

**
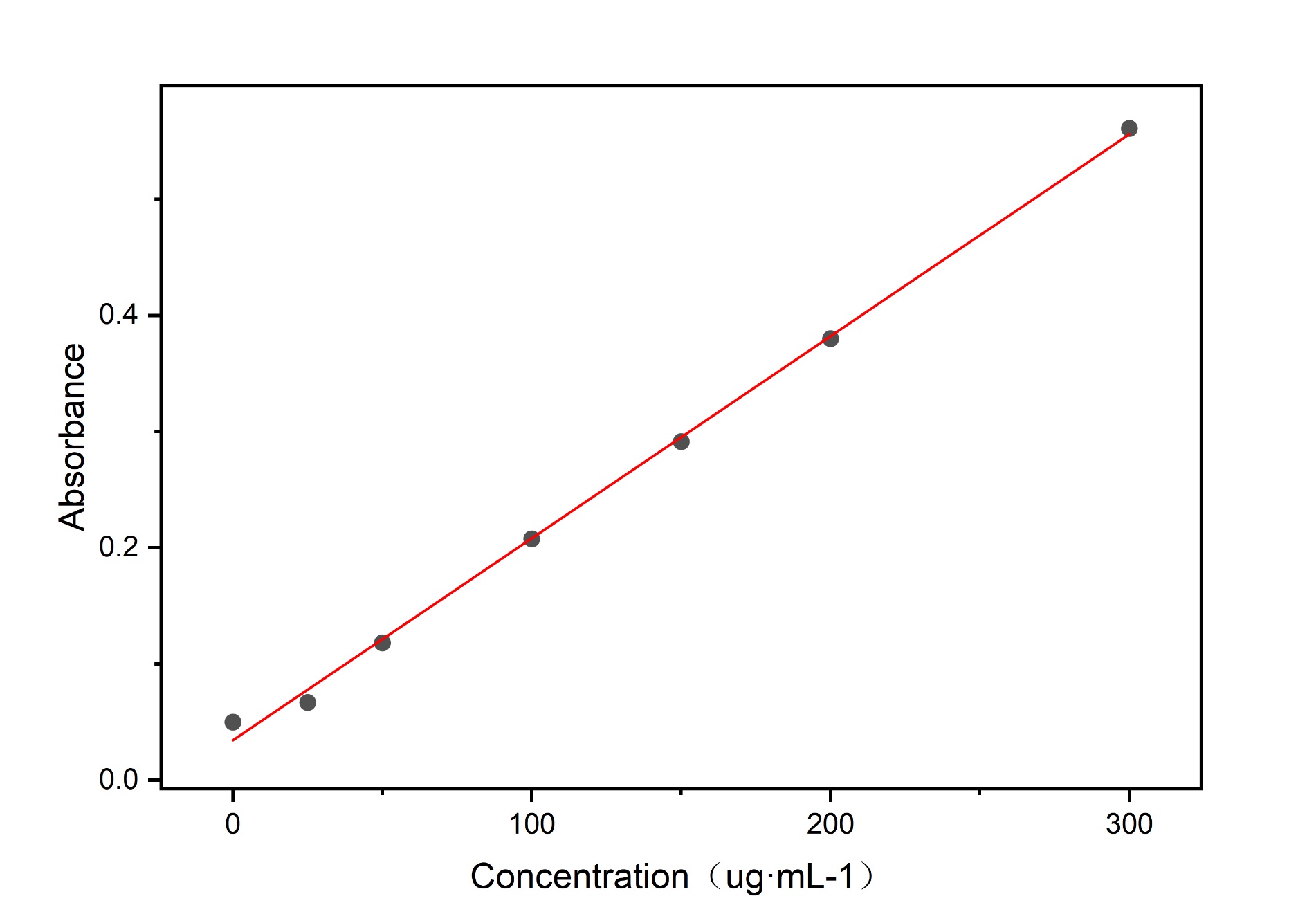
Fig.S11** Standard curve measured three times and averaged for linear fitting. The fitted equation is: y = 0.0344 + 0.0017x R^2^=0.998.

**Supplementary Figure 12.** Cumulative release curve of LBP**.**


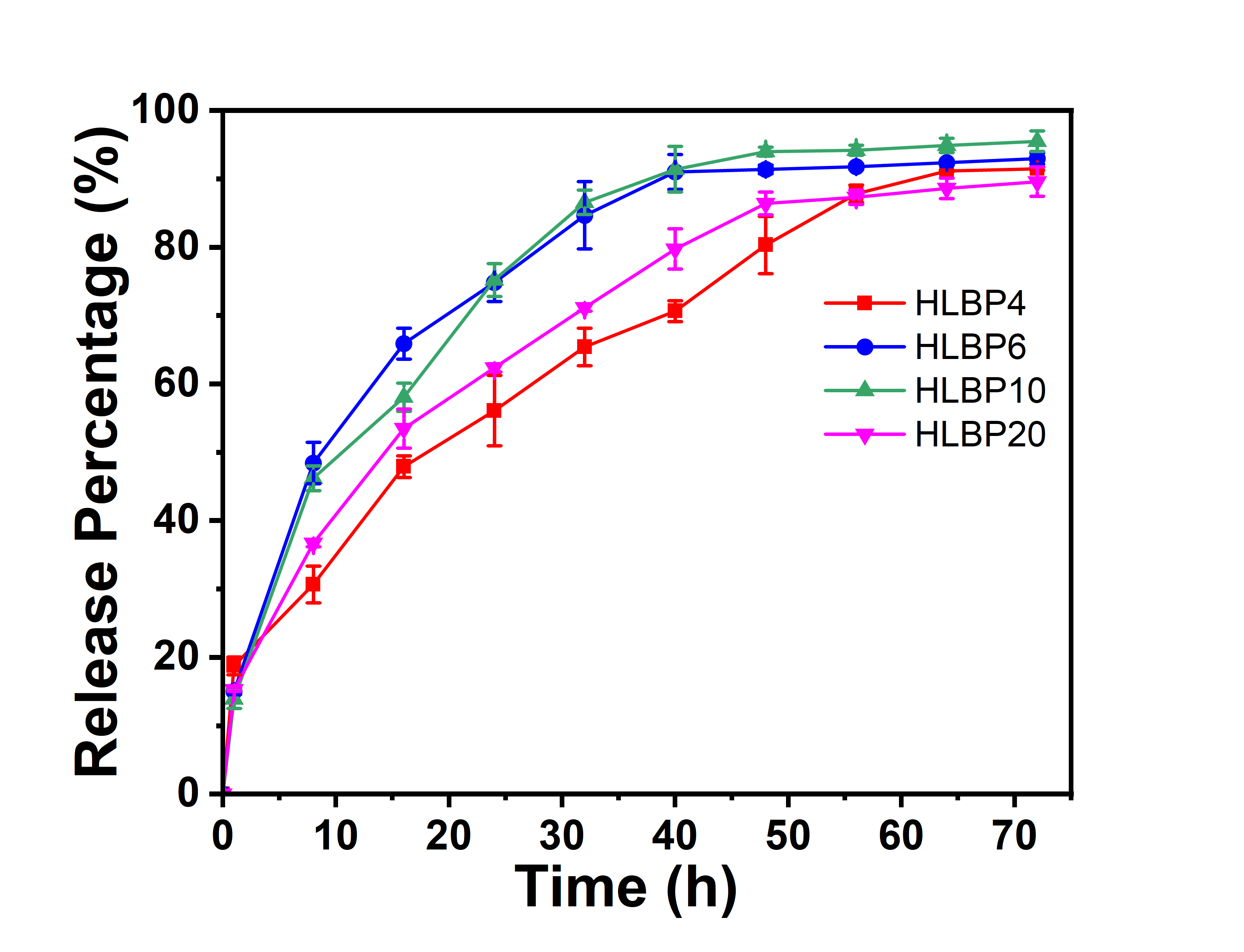


**Fig.S12** The cumulative proportion of LBP released from HLBP hydrogels. Statistical analysis was conducted based on n = 3 per group.

**LBP Release Test**

HLBP10 hydrogel was placed in a centrifuge tube containing 100 mL of PBS buffer (pH = 7.4). At specific time intervals (1 h, 8 h, 16 h, 24 h, 32 h, 40 h, 48 h, 56 h, 64 h, and 72 h), buffer solution samples were extracted, and the concentration of goji polysaccharides in the buffer was quantified using UV spectrophotometry. Finally, the LBP concentration was determined using the calibration curve equation, and a cumulative drug release profile over time was plotted.

To establish the standard UV curve for LBP, a 5 mg/mL LBP standard solution was prepared using PBS buffer. A full-wavelength scan (190–500 nm) was performed with a UV spectrophotometer to determine the maximum absorption wavelength of LBP. Subsequently, 5 μL, 10 μL, 20 μL, 30 μL, 40 μL, and 60 μL of LBP were each dissolved in 1 mL of PBS, and the absorbance at the maximum wavelength was measured to generate the standard curve (Figure S11).
